# Systematic review of the predictors of statin adherence for the primary prevention of cardiovascular disease

**DOI:** 10.1371/journal.pone.0201196

**Published:** 2019-01-17

**Authors:** Holly F. Hope, George M. Binkley, Sally Fenton, George D. Kitas, Suzanne M. M. Verstappen, Deborah P. M. Symmons

**Affiliations:** 1 Arthritis Research UK Centre for Epidemiology, Centre for Musculoskeletal Research, Faculty of Biology, Medicine and Health, The University of Manchester, Manchester. England; 2 School of Sport, Exercise and Rehabilitation Sciences, University of Birmingham, Edgbaston, Birmingham, England; 3 Rheumatology Department, Russells Hall Hospital, Dudley Group NHS Foundation Trust, Dudley, England; 4 NIHR Manchester Biomedical Research Centre, Central Manchester University Hospitals NHS Foundation Trust, Manchester, England; Leibniz Institute for Prevention Research and Epidemiology BIPS, GERMANY

## Abstract

**Introduction:**

Previous research has shown that statin adherence for the primary prevention of CVD is lower compared to secondary prevention populations. Therefore the aim of this systematic review was to review predictors of statin adherence for the primary prevention of CVD.

**Methods:**

A systematic search of papers published between Jan 1984 and May 2017 was conducted in PubMed, PsycINFO, EMbase and CINAHL databases. A study was eligible for inclusion if; 1) it was a study of the general population or of patients with familial hypercholesterolemia, hypertension, diabetes or arthritis; 2) statins were prescribed; 3) adherence was defined and measured as the extent to which patients followed their statin regimen during the period of prescription, and 4) it was an original trial or observational study (excluding case reports). A study was subsequently excluded if 1) results were not presented separately for primary prevention; 2) it was a trial of an intervention (for example patient education). Papers were reviewed by two researchers and consensus agreed with a third. A quality assessment (QA) tool was used to formally assess each included article. To evaluate the effect of predictors, data were quantitatively and qualitatively synthesised.

**Results:**

In total 19 studies met the inclusion criteria and nine were evaluated as high quality using the QA tool. The proportion of patients classed as “adherent” ranged from 17.8% to 79.2%. Potential predictors of statin adherence included traditional risk factors for CVD such as age, being male, diabetes and hypertension. Income associated with adherence more strongly in men than women, and highly educated men were more likely and highly educated women less likely to be adherent. Alcohol misuse and high BMI associated with non-adherence. There was no association between polypharmacy and statin adherence. The evidence base for the effect of other lifestyle factors and health beliefs on statin adherence was limited.

**Conclusion:**

Current evidence suggests that patients with more traditional risk factors for CVD are more likely to be adherent to statins. The implications for future research are discussed.

## Introduction

HMG-CoA (3-hydroxy-3-methyl-glutaryl-coenzyme) reductase is the rate-controlling enzyme of the mevalonate pathway, the metabolic pathway that produces low density lipoprotein cholesterol (LDLc). HMG-CoA reductase inhibitors (statins) reduce the level of LDL-C and other isoprenoids (lipids) and thereby lower the risk of atherosclerosis and cardiovascular disease (CVD) [1]. Statins also have multiple other (so called pleiotropic) effects which may contribute to the reduction of CVD risk including effects that stabilise atherosclerotic plaques, support endothelial function and reduce inflammation of the vasculature [2]

Whilst statins are used for the primary and secondary prevention of CVD the risk of death is lower for the primary prevention population, therefore it is important to evaluate the benefit of statins in this setting separately. Data from a prior meta-analysis of eleven randomised controlled trials (RCTs) indicated there was no association between statin use and all-cause mortality (RR 0.91, 95% CI 0.81–1.01) [3]. More recently, Taylor et al. conducted a Cochrane review of 18 RCTs and 19 observational studies and reported compared to placebo, statins reduced lipid levels and also the risk of experiencing a fatal or non-fatal cardiovascular event (CVE) by a quarter (RR 0.73, 95% CI 0.67–0.80), the equivalent number needed to treat (NNT) for five years was 56 (95% CI 46–75) [4]. They also analyzed the incidence of adverse events, including cancer, myalgia and rhabdomyolysis, arthritis, and increased liver enzyme, and found no evidence of increased risk for statin users compared to placebo or control participants, except in one trial in which there was an increased risk of type II diabetes [4]. The authors postulated there may be a risk of stroke, but there was no data to investigate this, and the authors recognized that not all trials in their review investigated side effects. Thus, there is evidence for cardiovascular but not wider benefits from statins use in the primary prevention setting.

Current UK clinical guidelines recommend that a person with at least a one in ten risk of experiencing a fatal or non-fatal CVE in the next ten years should be offered atorvastatin at 20mg daily or an equivalent dose of another licensed statin for its primary prevention [1]. The risk of primary CVD is calculated using a cardiovascular risk calculator. The QRISK3 calculator is most commonly used in the UK [5], the Framingham Risk Score (FRS) in the US [6], and the Systematic Coronary Risk Evaluation (SCORE) in Europe [7]. All of these calculators include the following components to calculate the risk of CVD; age, gender, smoking status, systolic blood pressure, the level of LDL-C and presence of co-morbid diabetes and hypertension, and some contain additional factors [8].

Once a clinician has prescribed statins, the extent to which these therapies will be effective is directly associated with the patient’s adherence to their treatment regimen [9]. A recent meta-analysis of rates of adherence in patient populations over 65 years of age revealed adherence to statins indicated for the primary prevention of CVD was suboptimal. At one year, only 47.9% were adherent and 24% had discontinued their therapy [10]. In 2013, a meta-analysis of 44 studies which investigated the relationship between statin adherence and mortality in primary and secondary populations found that 60% of 1,978,919 subjects were adherent. Adherence in this case was measured using pharmacy records and defined by calculating the ratio of the number of days that the patient had medication divided by the total number of days the patient was ‘observed’ (medication possession ratio (MPR)). An MPR ≥ 80% is classified as adherent. This level of adherence was associated with a reduced risk of all-cause mortality (RR 0.55, 95%CI 0.46–0.67) and CVD mortality (RR 0.85, 95%CI 0.81–0.89) which is equivalent to one CVD outcome per 10,000 individuals. The absolute risk is small, however it was calculated using the standardised death rate for people under the age of 65 and thus will be higher in older age-groups with higher baseline risks [11]. The greater reduction in all-cause mortality is supportive of a ‘healthy adherer’ effect, where adherence to statins is an indicator of other health promoting behaviours [12]. More recently, observational studies using registry data have reported a dose dependent relationship between increasing levels of adherence and reductions in cholesterol levels and CVD [13–15]. This relationship is apparent even at the highest levels of adherence; patients with 90–100% adherence (measured using MPR) were significantly more likely to have a reduction in LDL, high density lipoproteins and total cholesterol levels by at least 25% compared to those with 80–89% adherence [14]. In addition to the demonstrable health benefits associated with optimal statin use there are also economic benefits to high levels of adherence, and these cost benefits increase as the baseline risk of a primary CVE increases [16,17].

These studies highlight the need to optimise statin use for people at risk of a CVE. In order to improve adherence to statins in the primary prevention population, the predictors of and reasons for statin non-adherence need to be understood. Non-adherence can be intentional or unintentional. Intentional non-adherence refers to a person’s decision to take drug-holidays or stop the medication, and unintentional non-adherence includes forgetting or running out of medication. The extent to which non-adherence occurs is related to the cognitive, emotional and financial resources of the patient, and their healthcare context [18]. Systematic reviews of other long-term medications have identified psychological factors such as mood, treatment beliefs and coping strategies as important predictors of adherence. Qualitative research with a primary prevention cohort found that reasons for intentional statin non-adherence or discontinuation included perceived side effects and the inflexibility of the healthcare provider to switch statins [19].

Previous systematic reviews have focussed on statin non-adherence rather than adherence and identified the following risk factors for non-adherence; high cost, low income, absence of co-morbidities, infrequent lipid monitoring, high intensity dosing and being an incident user versus existing user of statins [20–23]. Importantly, these reviews included both primary and secondary prevention population studies in their analyses and the prescription of statins for the primary prevention of CVE was shown to have the largest pooled effect size on the risk of non-adherence. A recent study demonstrated that patients identified as statin non-adherent prior to their first CVE were less likely to be non-adherent post hospitalisation, which illustrates the difference between primary and secondary prevention populations with respect to adherence [24]. The evidence suggests thus far that the primary prevention population appears to be at greater risk of non-adherence. Given the negative consequences of non-adherence in this population with respect to increased risks of CVD, identifying the risks of statin non-adherence specific to the primary prevention setting is merited. Lemstra et al. lamented the lack of studies investigating the effect of psychological and lifestyle factors on adherence [20]. Factors such as depression, medication and illness beliefs have been shown to predict non-adherence to other long-term medications [25]. A contemporary meta-analysis of interventions to improve statin adherence found strategies such as patient education, counselling, simplifying regimens, issuing reminders and even interventions classed as multi-faceted that included a combination of the above strategies only achieved small positive effects upon adherence (Hedges g<0.5) [26]. Clearly there is a need to investigate the reasons for and predictors of adherence in more depth as this will allow for better targeted and tailored strategies to optimise statin adherence for the primary prevention of heart disease.

Therefore the aim of this systematic review is to identify predictors of statin adherence for the primary prevention of CVD.

## Methods

### Search strategy

EMbase, Medline, CINAHL (Cumulative Index to Nursing and Allied Health Literature) and PsycInfo databases were searched from January 1984 (when the first trials of statins were published) until May 2017 [27], using Patient Intervention Comparison Outcome (PICO) search methodology to build the following strategy [28]: P)Primary prevention as the patient population; I) one or more statins as an intervention; C) predictors of adherence as comparators and O) adherence as a measured study outcome. The PICO comparison category would be identified at subsequent stages of the selection process. Synonyms for each PICO category were defined and the databases searched to identify abstracts that included a synonym from each category in the title, original title, abstract, subject heading, name of substance, or registry word fields (S1 Table). The apriori review protocol is available upon request.

### Study inclusion

Studies obtained from the systematic search were eligible for inclusion if: 1) People were receiving treatment for the primary prevention of CVD or the results were given separately for primary prevention; 2) a statin was prescribed; 3) adherence was defined as the extent to which patients followed their statins regimen during the period of prescription, rather than the length of time till statin discontinuation; 4) predictors of adherence were defined and measured and 5) the study was a piece of original research (including abstract, thesis or conference proceedings). Titles and abstracts obtained from the search were independently evaluated by two researchers HH, SF and, where there was a disagreement, adjudicated by a third reviewer (DS). If the cohort was not defined as primary or secondary prevention cohort, for example if registry or pharmacy refill datasets were used to create ‘incident statin user’ cohorts, the reviewers assumed these analyses would include at least some incident statin use after a CV event or diagnosis and therefore these studies were excluded from the review. If primary and secondary prevention populations were jointly investigated, studies were only included if results specific to the primary prevention sample were evident. If the number or proportion of patients in adherent and non-adherent groups were presented these data were extracted and odds ratios for adherence calculated. Trials were included if there were secondary analyses of both arms of the study that investigated predictors of adherence.

If original research met the inclusion criteria but only existed as an abstract, thesis or conference proceedings, and the effect of factors on adherence was available it was included. Relevant reviews and opinion articles were retrieved in order to cross reference to ensure all relevant articles were included.

### Quality assessment

The quality of the included studies was formally assessed using the quality assessment tool measure used in a previous published systematic review by Hope et al [25]. The quality assessment consisted of sixteen items, adapted from the recommendations of Sanderson et al. which state that observational studies should be evaluated on the use of appropriate methods to: 1) select participants, 2) measure exposure and outcome variables, 3) control for confounding, 4) reduce bias and 5) analyse data [29] (S2 Table). The authors judged papers that scored fourteen or more as high quality (range (0–17)). Trials were judged using the same criteria, since the data were analysed as if they were prospective cohort studies.

### Evidence synthesis

#### Quantitative synthesis

Rates of non-adherence were inverted to calculate a rate of adherence for each study. A predictor was selected for quantitative pooling if there were at least three studies with a combined sample ≥1000 that investigated the same or similar predictor using the same or similar analysis (binary versus continuous data). Where cohorts were stratified by age, the effect size and the sample size of said strata were entered as separate effects into the meta-analysis. Where studies had stratified their cohort and obtained separate estimates for each cohort these were treated as separate cohort studies. Where studies had used the same data source the study with the larger sample size was included in the meta-analysis unless the effect was only investigated in the smaller study. These estimates were pooled using a fixed effects meta-regression analysis that adjusted for the study sample size. The I^2^ statistic was used to evaluate the proportion of variance across the studies attributable to study heterogeneity. Sensitivity analyses of adjusted and unadjusted effect sizes, period of follow-up (≤ 1 year, 1 year, > 1 year), region, gender distribution, age range, measure of adherence and % adherent (< 50% versus ≥ 50%) were conducted to identify possible sources of heterogeneity.

#### Qualitative synthesis

Predictor data that did not meet criteria for quantitative synthesis, or data where the pooled estimate possessed high heterogeneity (I^2^>50%), were qualitatively compared across studies and evaluated based on the definitions of strong, moderate, limited and conflicting evidence of van Tulder and colleagues [30]. Strength of evidence for an association was graded 1–5, where 5 meant there were multiple high quality studies, where high quality meant the study scored ≥14 on the QA score and the specific analysis adjusted for potential confounding, with a total sample size ≥1000. To score four there had to be a total sample size ≥1000 from several studies including one high quality study. To score 3 there had to be evidence from one high quality study, or several low quality studies with a total sample size ≥1000. To score 2 the evidence was taken from several low quality studies or one high quality study with a total sample <1000, and 1 was scored where there was only one low quality study with a sample less than 1000. Where there were inconsistent findings with the same level of evidence these were classed as ‘0’ to indicate conflicting evidence. The evidence could be conflicting in relation to the presence of or direction of an effect (Table 1). All unique predictors were included in the qualitative synthesis. Where studies utilised the same cohorts and duplicate effects existed then the effect size from the higher quality study was included in the synthesis.

**Table 1:** Quality criteria for strength of evidence and conflicting evidence.

Strength of evidencea	1	2	3	4	5	0
Quality	1 low quality study	Several low quality studies or 1 high quality study	1 high quality study or Several low quality studies	1 high quality study and Several low quality studies	Several high quality studies	Equivalent strength of evidence (1–5) for the presence or direction of effect.
Requirement for adjustment	No	No	No	Yes	Yes	NA
Sample size	Total sample < 1000	Total sample < 1000	Total sample ≥ 1000	Total sample ≥ 1000	Total sample ≥ 1000	NA

## Results

**Fig 1:**
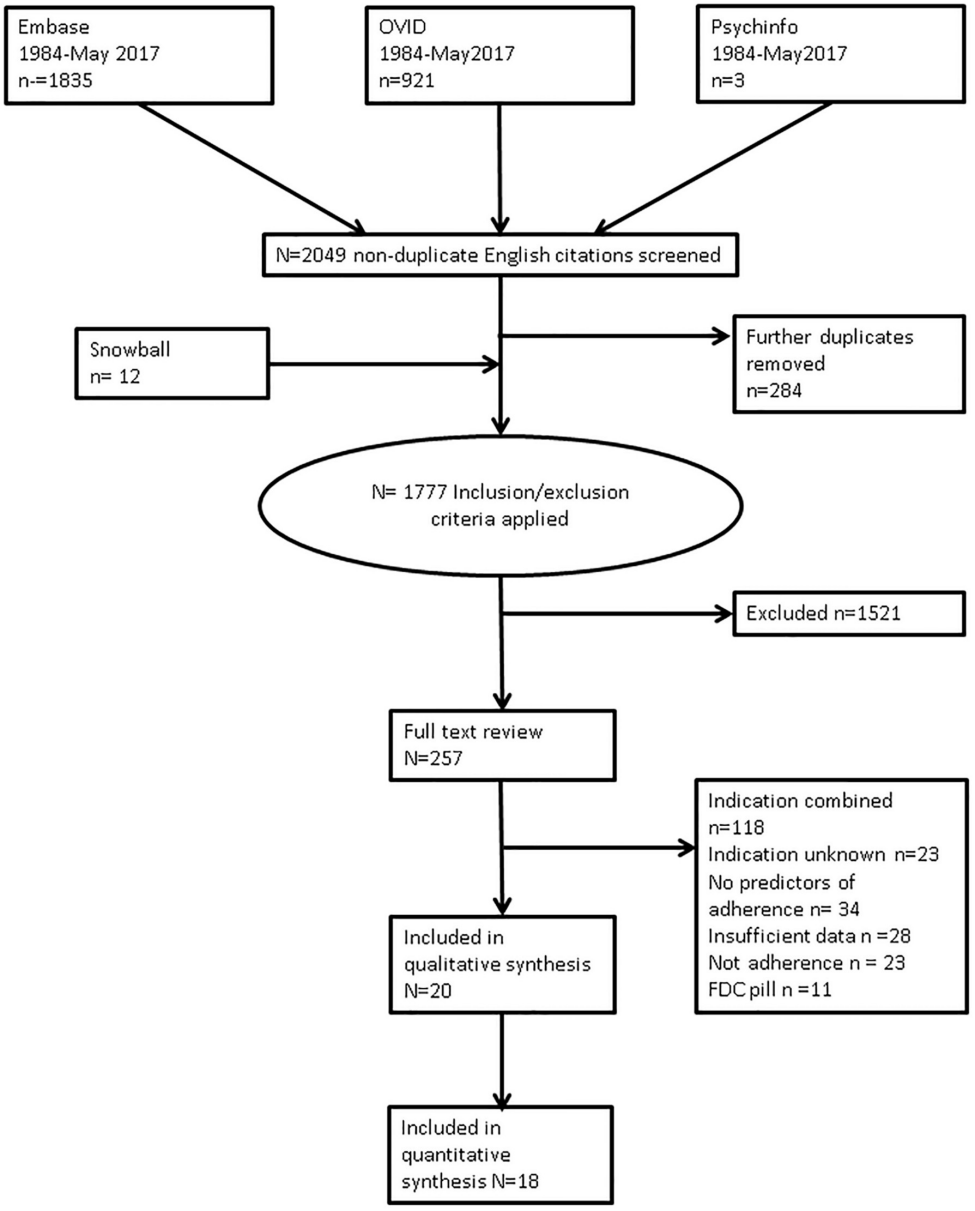
PRISMA flow diagram of article selection process.

The systematic search generated 2049 abstracts, a further 12 were included after snowballing and after duplicates (n = 284) were removed. 1777 abstracts were screened. After screening 257 abstracts fulfilled the inclusion criteria and a full paper review was performed. Some papers had to be excluded because the patient cohort was not clearly defined (n = 23) or the primary and secondary prevention cohorts were analysed together (n = 118). Other papers were excluded because variables were not compared across adherence levels (n = 34), or there was insufficient data to include (n = 28), discontinuation or persistence were measured rather than adherence (n = 23), or they were fixed dose combination (FDC) therapies (n = 11). A total of 19 papers fulfilled all inclusion criteria (Fig 1).

### Study characteristics

The review consists of three cross-sectional studies [31–33], eleven retrospective cohort studies [34–44], three prospective cohort studies [45–47] and two randomised controlled trials (RCTs) [48, 49] (Total n = 19). Of the retrospective studies, one was stratified by gender [34], one by age and gender [44] and one by length of follow-up [36]. The included RCTs contained analyses which combined patients from treatment and comparator arms that investigated the association between patient factors and adherence [48,49]. Nine studies were judged to have a quality assessment score of fourteen or more and to have adequately controlled for potential biases in their study design and planned data analyses [32,34–36,38,39,44,46,47]. Adherence was not the primary outcome in six studies [37,39–43], only seven adjusted for the effect of other variables upon adherence [31,34–36,38,44,46]. Ten studies used statin refill data extracted from drug registries and calculated the proportion of days covered (PDC) or a MPR [34–42,44–47]. Adherence was electronically monitored in one study and a composite measure of adherence to the dose and schedule was calculated [49]. Five studies used self-report measures; some of which were validated [31,32,47]; the remainder used bespoke self-report measures [33,48]. Adherence was assessed over time periods as brief as one month [33] to five years [37]. Most studies investigated predictors of being adherent, where being adherent was defined as adherence ≥80%. Specific cut-offs on self-reported measures were used to define being adherent or endorsement of the adherent behaviour (e.g. yes, I am adherent). In one study no-one self-reported high adherence so a cut-off of moderate adherence was used [31]. One study did not include predictors but it did contain reasons for non-adherence so it remained in the review [33]. Only one study investigated the predictors of adherence using a continuous adherence outcome [49].

**Fig 2:**
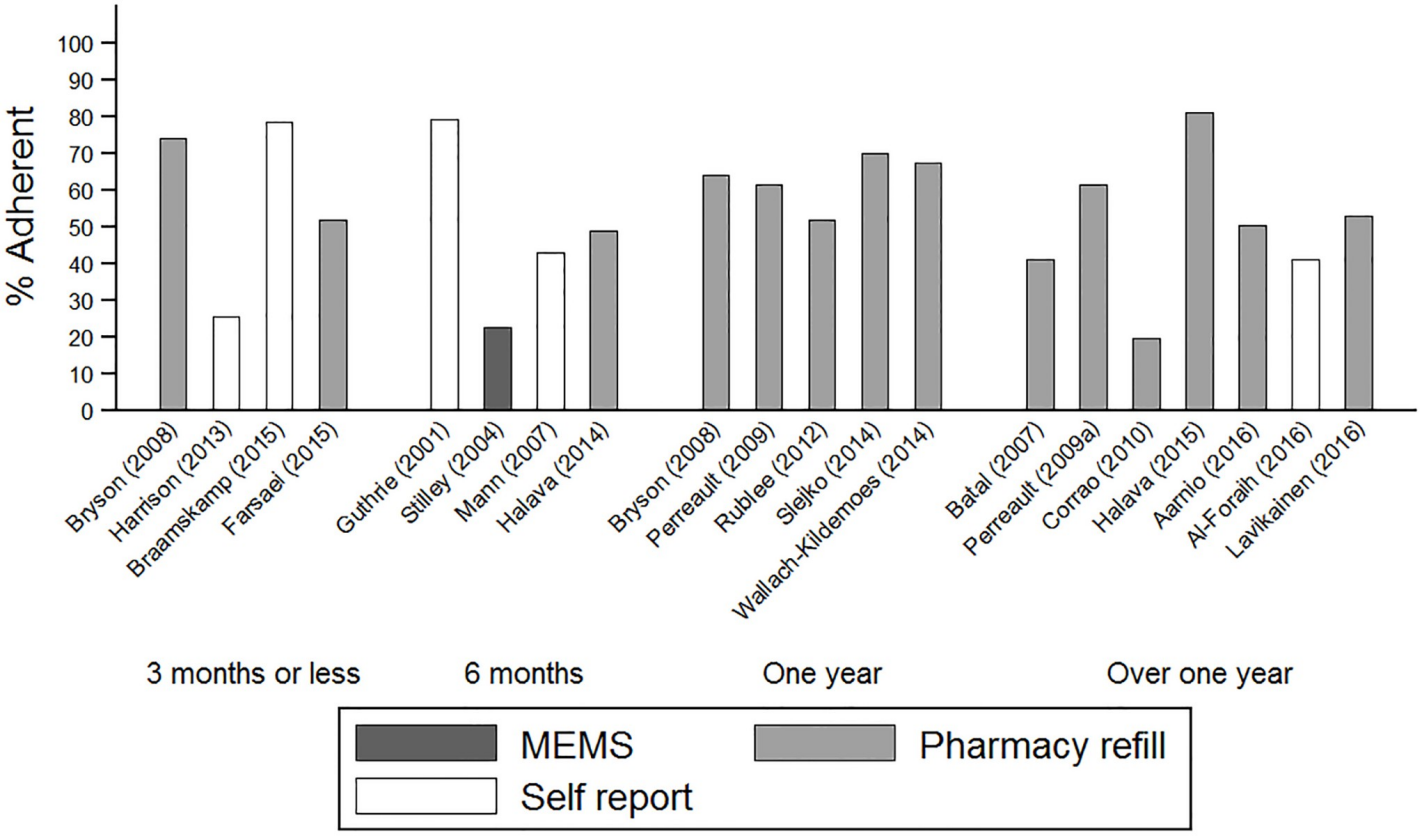
Percentage of patients adherent to statins grouped by follow-up and adherence measure. MEMS; Medcation event monitoring system, Pharmacy refill; medication possession ratio (MPR) or Proportion of days covered (PDC).

The proportion of patients included in the review defined as adherent ranged from 17.8% to 79.2%, which indicates that overall adherence to statins for the primary prevention of CVD, however it was measured, appears to be sub-optimal. There was some evidence that the wide variation of the number of adherent patients reflects the heterogeneity across studies with respect to the characteristics of the sample and study design (Table 2). The length of follow-up and type of adherence measure appeared to account for some of the variability observed across studies (Fig 2). Quantitative synthesis of the data that met the conditions for a meta-analysis revealed pooled estimates with high heterogeneity (I^2^>90%), therefore only the qualitative synthesis is reported.

**Table 2:** Description of studies included in the review.

Study	Population/ Country	Adherence measure	Design	N	Age (years)*	% female	Adherence definition	Follow-up	% adherent	QA score	Adjusted ES
**Stilley (2004)**[49]	Volunteer/ USA	MEMS dose & schedule	RCT	158	46.2 (8.7)	46.2	≥ 80%	6 months	22.8	10	no
**Farsaei (2015)**[45]	Diabetes / Iran	MPR	Prospective Cohort	158	56.4 (9.3)	66.4	≥ 80%	3 months	51.8	7	no
**Halava (2014)**[46]	Population register / Finland	PDC	Prospective Cohort	6458	24–75	77.9	≥ 80%	6 months	49.1	16	yes
**Batal (2007)**[35]	HMO register / USA	MPR	Retrospective cohort	3292	57.8 (10.9)	57.1	≥ 80%	1.5 years	41	13	yes
**Bryson (2008)**[36]	HMO register / USA	PDC	Retrospective Cohort	5473	64 (9.7)	2.4	≥ 80%	3 months	74	14	yes
		PDC					≥ 80%	1 year	64	14	yes
**Perreault (2009)**[40]	HMO register / Canada	MPR	Retrospective Cohort	242914	45–85	58	≥ 80%	1 year	61.6	13	no
**Perreault (2009a)**[41]	HMO register / Canada	MPR	Retrospective Cohort	55134	45–85	60	≥ 80%	3 years	61.6	13	no
**Corrao (2010)**[37]	HMO register / Italy	PDC	Retrospective cohort	90832	61.8 (11.1)	59.3	≥ 80%	5 years	19.6	13	no
**Rublee (2012)**[42]	HMO register / USA	PDC	Retrospective Cohort	79010	NP	46	≥ 75%	1 year	51.9	13	no
**Slejko (2014)**[43]	Population register / USA	PDC	Retrospective Cohort	11126	55.9 (10.3)	46.6	≥ 80%	1 year	70.2	13	no
**Wallach-Kildemoes (2014)[44]**	Population register / Denmark	PDC	Retrospective Cohort	26397	40–64	100	≥ 80%	1 year	69.2	16	yes
		PDC		24886	40–64	0	≥ 80%	1 year	63.8	16	yes
		PDC		8765	65–84	0	≥ 80%	1 year	67.9	16	yes
		PDC		15990	65–84	100	≥ 80%	1 year	69.2	16	yes
**Halava (2015)**[38]	Population register / Sweden	PDC	Retrospective cohort	5033	44–68	0	≥ 80%	4 years	82.2	15	yes
		PDC		4232	44–68	100	≥ 80%	4 years	78.3	15	yes
**Aarnio (2016)**[34]	Population register / Finland	PDC	Retrospective cohort	116846	60.8 (7.8)	100	≥ 80%	1.5 years	50.5	16	yes
		PDC		51590	58 (7.7)	0	≥ 80%	1.5 years	51.3	16	yes
**Lavikainen (2016)**[39]	Population register / Finland	PDC	Retrospective Cohort	42807	55–59	100	≥ 80%	1.5 years	53	13	no
**Guthrie (2001)**[48]	Primary care research register /USA	Self-report	RCT	4548	58.0 (NP)	52.4	“Yes”	6 months	79.2	5	no
**Mann (2007)**[47]	Veterans/ USA	Self-report	Prospective Cohort	71	61 (12.6)	10	MAS <11	6 months	43	14	no
**Harrison (2013)**[33]	HMO register / USA	Self-report	Cross-sectional	98	59.3 (13.4)	46.9	Filled 1^st^ prescription	3 months	25.5	4	no
**Braamskamp (2015)**[32]	Familial hypercholesterolemia/ Netherlands	Self-report	Cross-sectional	169	24 (3.2)	54	MASRI VAS ≥80	1 month	78.7	14	no
**Al-Foraih (2016)**[31]	Hypercholesterolemia/ Kuwait	Self-report	Cross-sectional	200	51–60	68.5	MMAS score ≥ 6	NA	41	13	Yes

*Mean(SD) otherwise range; HMO; Health Maintenance Organisation; RCT = Randomised Controlled Trial; PDC = Proportion of days covered; MPR = Medication Possession Ratio; MEMS = Medication Event Monitoring System; MMAS-8; Morisky Medication Adherence Scale MASRI VAS; Medication Adherence Self-Report Inventory Visual Analogue Scale; MAS: Morisky Adherence Scale; QA score = Quality Assessment Score; ES = Effect size.

### Traditional cardiovascular risk factors

The standard components of the CVD risk scores (i.e. age, male gender, LDL-C levels, the presence of comorbid diabetes and the presence of co-morbid hypertension) are used by physicians to decide who should be prescribed a statin for the primary prevention of a CV event. We considered these patient factors as potential predictors of adherence because the physician may make the patient aware that possessing these characteristics increases their ten year risk for a CV event.

#### Age-positively associates with statin adherence (Strength of evidence = 5)

**Fig 3:**
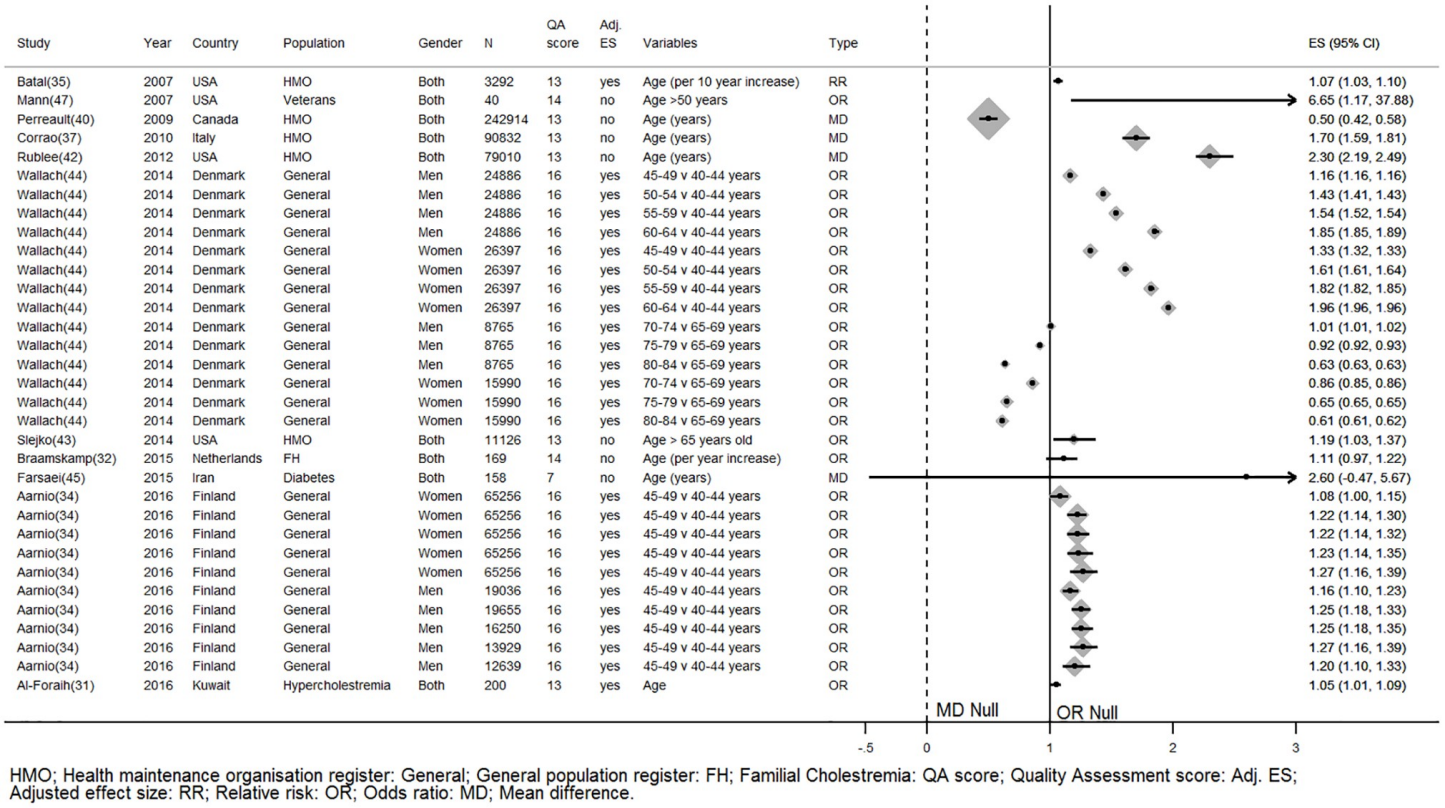
The relationship between age and statin adherence. *Wallach-Kildemoes; HMO: Health maintenance organisation; General: General population register; FH: Familial hypercholesterolemia; HC: Hypercholesterolemia; QA: Quality assessment; Adj. ES: Adjusted effect size; RR: Relative risk; OR: Odds ratio; MD: Mean difference.

There is strong evidence that older age predicts statin adherence. Four studies (three high quality & one low quality), including a total of 662638 participants, that adjusted for confounders [31,34,35,44] and six studies (Total N = 496921, two high quality & four low quality) with unadjusted effects found adherence increased with older age [37,40,42,43,47]. One small (N = 169) high quality study and one small low quality study (N = 158) found age did not associate with adherence [32,45] (Fig 3). Braamskamp et al may have found a different effect because they investigated adherence in a cohort of young adults with familial hypercholesterolemia; this population has an average age of 24 years, this is much younger than the typical population who commence statins [32]. Wallach-Kildemoes et al. found that the adjusted odds of adherence increased by up to a factor of 2 per five year increase in age in their male and female cohorts aged 40 to 65 years [44]. From the same cohort study the odds of being adherent decreased by up to 60% per five year increase in age in the male and female cohorts aged 65–80 years.

#### Men are more adherent than women—Strength of evidence = 4

**Fig 4:**
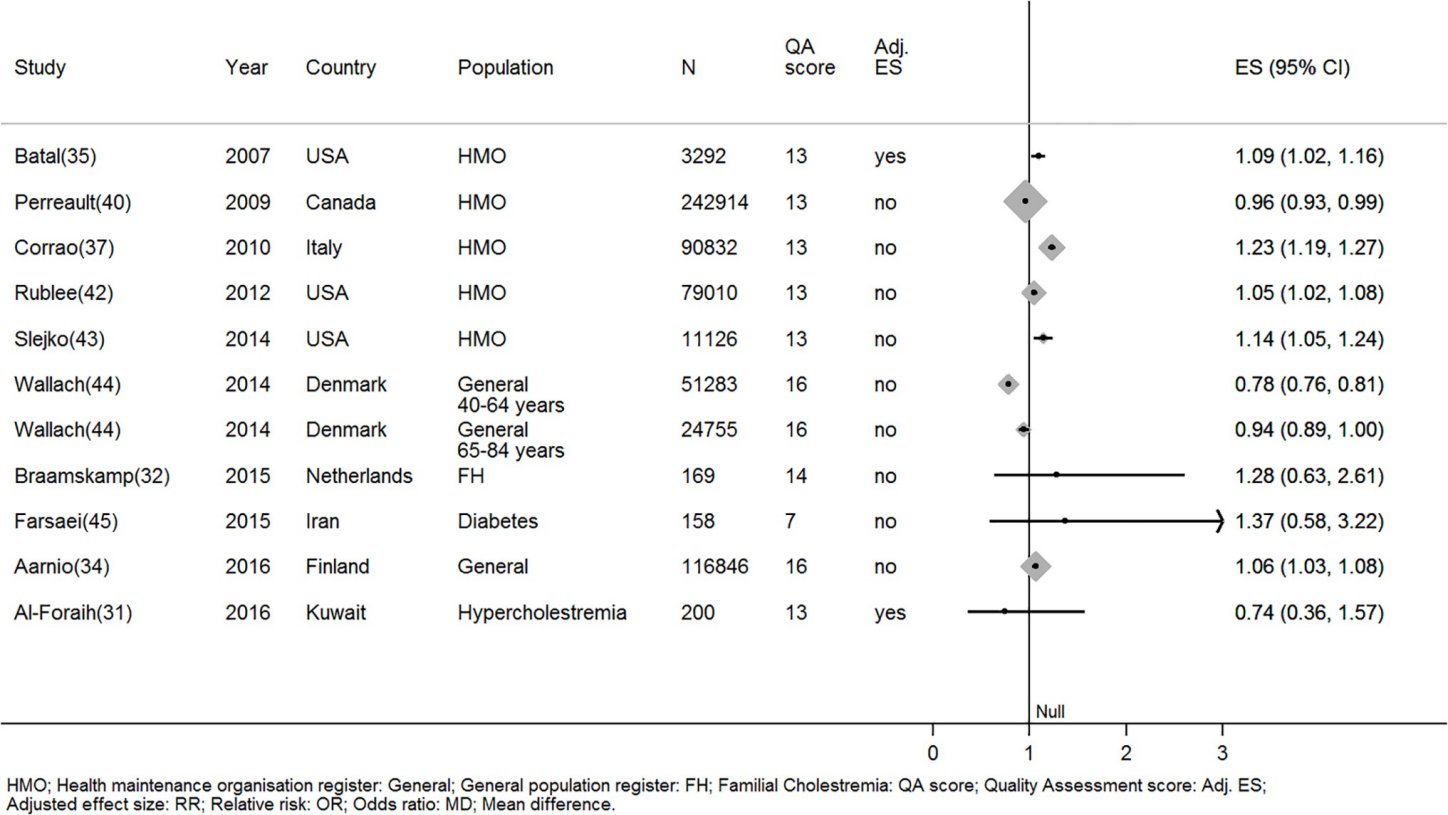
The relationship between being male and statin adherence. *Wallach-Kildemoes; HMO: Health maintenance organisation; General: General population register; HC: Hypercholesterolemia; QA: Quality assessment; Adj. ES: Adjusted effect size; RR: Relative risk; OR: Odds ratio.

One large high quality study that adjusted for other factors and four low quality studies with unadjusted effect sizes (Total N = 301106), reported men were more adherent than women [34,35,37,42,43]. In one high quality and one low quality study women were more adherent than men (Total N = 318952), but these effects were not adjusted for other factors [40,44]. Finally there was limited evidence that gender has no effect upon statin adherence from three studies (Total N = 527, n high quality = 1) [31,32,45](Fig 4).

#### High cholesterol / Dyslipidemia does not associate with statin adherence—Strength of evidence = 3

In one large high quality general population study the presence of dyslipidemia was not associated with the odds of being adherent in the female or male cohorts [32]. Braamskamp et al. found, in a population of young adults with familial hypercholesterolemia, baseline LDL-C levels were not associated with self-reported adherence over the past month (OR 0.90, 95%CI;0.70–1.19).

#### Diabetes associates with statin adherence—Strength of evidence = 4

**Fig 5:**
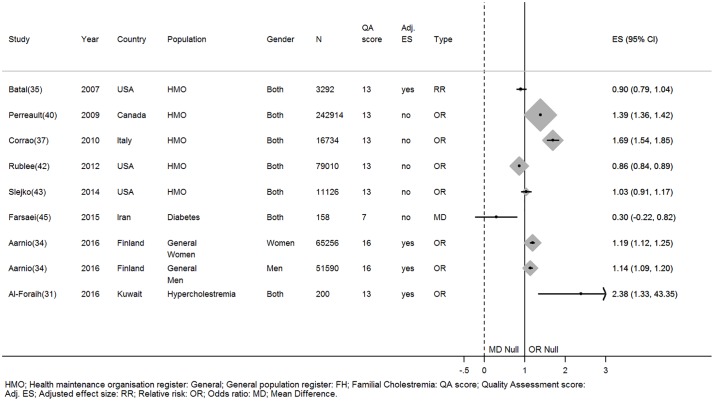
The relationship between diabetes and statin adherence. HMO: Health maintenance organisation; General: General population register; HC: Hypercholesterolemia; QA: Quality assessment; Adj. ES: Adjusted effect size; RR: Relative risk; OR: Odds ratio; MD: Mean difference.

Eight studies examined the relationship between diabetes and being adherent [31,34,35,37,40,42,43,45]. There was strong evidence that that people with diabetes are more likely to adhere to statins, four large studies including Aarnio et al. that adjusted for other confounders found that people with diabetes or who used of antidiabetic medications had an increased odds of being adherent compared to non-diabetics (Total N = 376694). There was moderate evidence that diabetes does not associate with adherence from three low quality studies (Total N = 14576, mean QA score = 11) and limited evidence that diabetes reduced the odds of being adherent (Total N 79010, QA score = 13) (Fig 5).

#### Hypertension/blood pressure associates with adherence—Strength of evidence = 5

**Fig 6:**
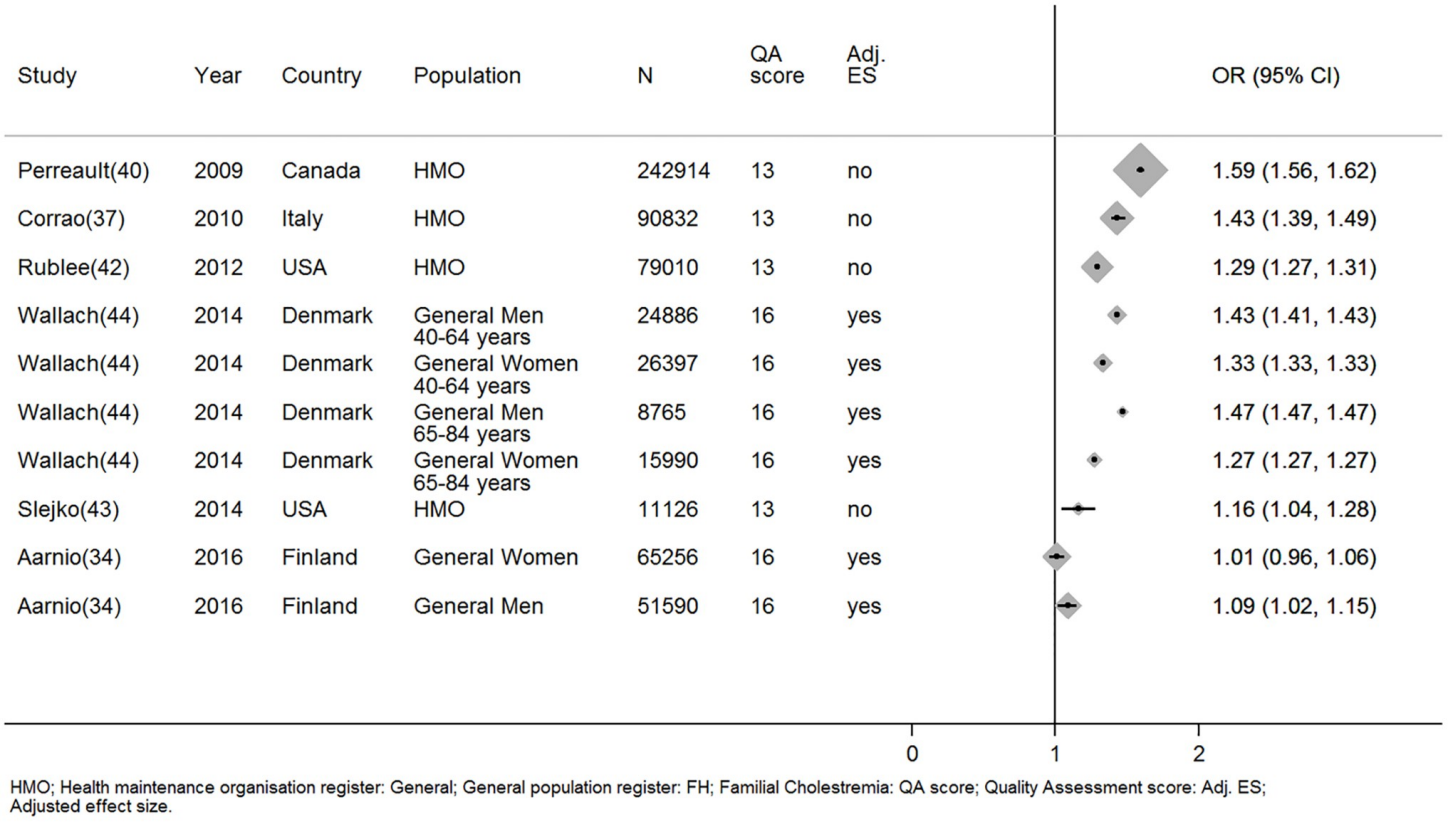
The relationship between Hypertension and statin adherence. *Wallach-Kildemoes; HMO: Health maintenance organisation; General: General population register; QA: Quality assessment; Adj. ES: Adjusted effect size.

Qualitative synthesis of the evidence indicated that there is very strong evidence that hypertension positively associates with statin adherence even after adjusting for other factors (Total N = 551094, n High quality = 2) [31,34,37,40,42], there was moderate evidence of no effect of hypertension on statin adherence in women (Total N = 65256, QA score = 16) [34], and no evidence of a negative effect (Fig 6). Aarnio et al. (2016) also noted that the odds of adherence to statins increased for every additional class of CV medications prescribed, and this effect was observed in both men (OR 1.03, 95%CI; 1.0–1.06) and women (OR 1.04, 95%CI;1.01–1.08).

#### Being an ex-smoker associates with adherence—Strength of evidence = 3

Smoking status was investigated in two studies. In one large high quality study being a current smoker (vs being a non-smoker) did not predict being adherent (OR 1.01 (0.86–1.18)) (n = 6458, QA score = 16), whilst in this same study being an ex-smoker predicted good adherence (OR 1.20, 95%CI;1.0–1.3) [46]. One small low quality study compared current smokers versus non-smokers and former smokers grouped together and found a non-significant negative effect of smoking (yes v no) (OR 0.69 95%CI: 0.23–2.07) (n = 200, QA score = 13) [31].

### Socioeconomic factors

Low socioeconomic status indicated by lower levels of income, education and work status are known to associate with CVD. One of the mechanisms through which this association may occur is lower levels of adherence to medications such as stains.

#### Higher income associates with adherence and interacts with gender—Strength of evidence = 5

Two high quality studies that adjusted for other confounders including socioeconomic factors compared adherence across income quintiles [34,44]. Wallach-Kildemoes et al. (2014) stratified their cohort by age and gender and then split their samples into quintiles of income that took into account family composition; the exact income thresholds for each quintile were not presented. Compared to participants in the lowest income quintile, participants in the higher income quintiles were more likely to be adherent, after adjustment for age, income, education and hypertension. This effect was observed in men and women of middle and post-retirement age; the strongest effects were observed for men of middle age (OR 1.56, 95%CI; 1.54–1.56) [43]. Aarnio et al (2016) used the taxable income per year to calculate income quintiles and used the wealthiest quintile as their reference category [33], in men there was a strong positive effect of income on the odds of adherence; compared to men in the wealthiest quintile men in succeeding lower income quintiles were less likely to adhere and the strongest effect was observed with men in the poorest quintile (OR 0.74, 95%CI; 0.68–0.79). The strength of these associations was attenuated in the cohort of women; only women in the poorest quintile were less likely to be adherent compared to women in the highest income quintile (OR 0.93, 95%CI; 0.86–1.00). These analyses adjusted for other socioeconomic and clinical factors but not smoking status.

#### Higher level of education associates with statin adherence and interacts with gender—Strength of evidence = 5

**Fig 7:**
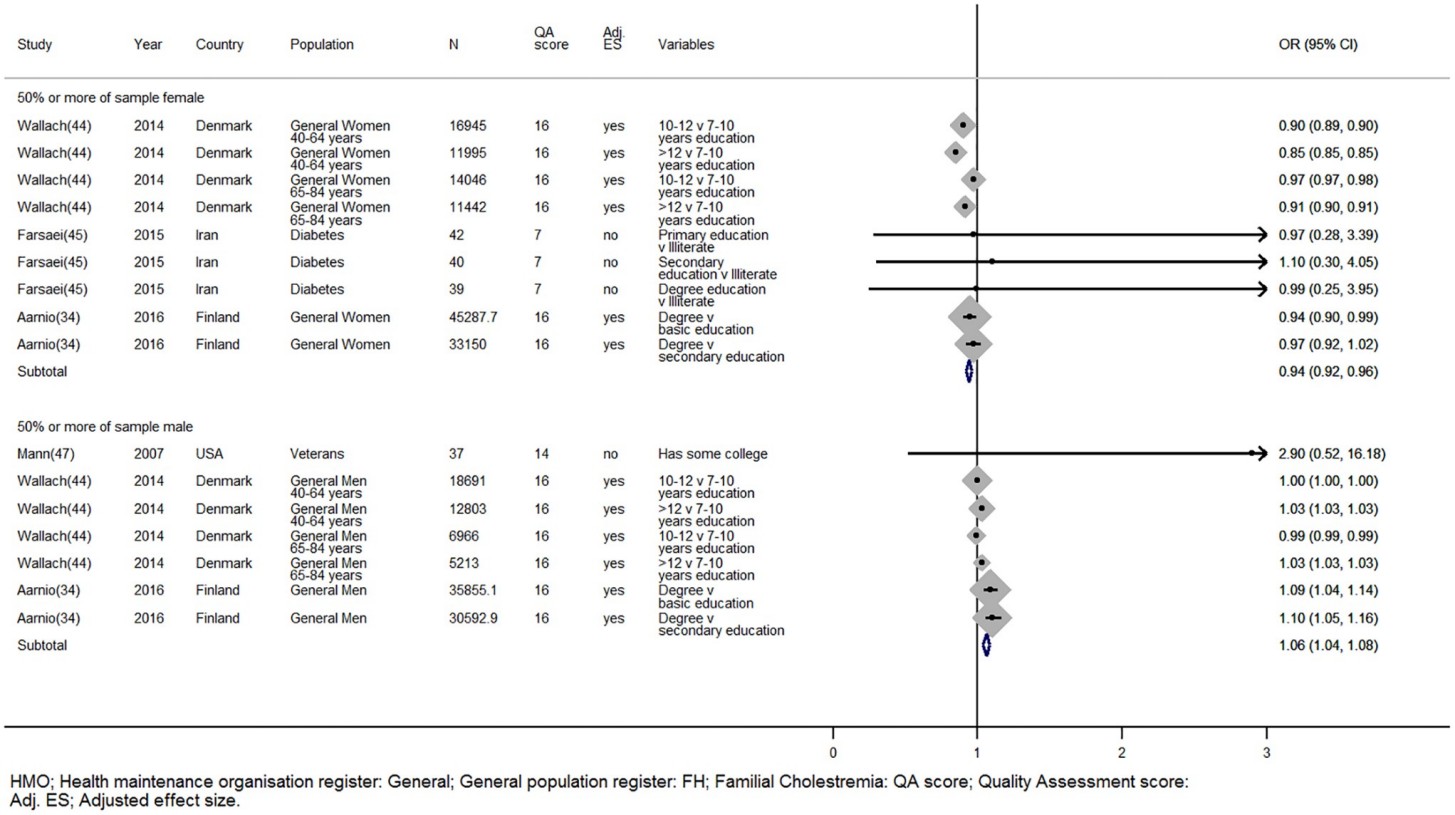
The sex dependent relationship between education and statin adherence. *Wallach-Kildemoes; HMO: Health maintenance organisation; General: General population register; QA: Quality assessment; Adj. ES: Adjusted effect size; RR: Relative risk; OR: Odds ratio.

Four studies provided data on the level of education and statin adherence [34,44,45,47]. In studies where more than 50% of the sample were male a higher level of education increased the likelihood of adhering (OR 1.07, 95%CI;1.04–1.10), whereas in studies where 50% or more of the sample were women a good education reduced the likelihood of adhering to statins (OR 0.92, 95%CI; 0.89–0.95). These estimates included two studies that were of high quality and adjusted for other confounders including socioeconomic factors [34,44]. Aarnio et al. found that the likelihood of being adherent was lower for men if they had a basic level or secondary level education compared to those with a degree [33]. Wallach-Kildemoes et al reported a similar positive effect in men who had 12 or more years of education compared to those with 7–10 years or 10–12 years of education [43]. This effect attenuated once income and age were controlled for in the analyses but remained for the most versus the least educated men. The opposite effect was observed in women; increasing levels of education were associated with a lower odds of being adherent, and these effects remained even after controlling for other covariates, the strongest effect was observed in Finnish women aged 40–64 years (OR 0.85, 95%CI; 0.85–0.85) (Fig 7).

#### The relationship between work status and statin adherence—Strength of evidence = 0

Two large high quality studies and one small low quality study investigated the effect of work on adherence [31,34,38]. Being in work reduced the likelihood of adhering to statins. In particular compared to employed people, retired people were 11% more likely to be adherent, and this appears to be the case for both men and women after adjustment for other factors including age and other comorbidities [34]. However, Halava et al. followed a Swedish cohort over the transition from employment to retirement and captured the prevalence of non-adherence [38]. Using a repeated measures design they found that adherence to statins was lower after retirement (PR 0.85, 95%CI; 0.80–0.88). Halava et al. adjusted for the calendar year, time in study, and age at retirement, but not for other factors such as the number or type of co-morbidities that may have confounded this relationship. Given there were only two studies with conflicting findings further research is needed. Simple comparisons of retired and employed groups should be avoided since they may be confounded by age, which strongly associates with both statin adherence and retirement.

#### The effect of region on adherence—Strength of evidence = 4

Four studies provided data on adherence across different regions including one large high quality study that adjusted for confounders and several studies with unadjusted effects Several studies looked at adherence in regions within countries and observed significant differences across regions of the USA, Finland and Kuwait [34,42,43,45]. The effect sizes observed suggest the effect of living in a particular place could reduce the odds of adhering by between 10 to 50% (Table 3). The reason for these differences could be variations in health, health services or socioeconomic factors. Without further knowledge of the economic and health profiles of these regions these data are difficult to interpret.

**Table 3:** Study characteristics and the investigated predictors of the included articles ordered by study design.

Reference (Year) Country	Covariates in multivariable models	Factors associated with good adherence	Effect size (Confidence Interval)	Factors not associated with good adherence
**Cross sectional**				
Al-Foraih & Somerset (2016) [31] Kuwait	Age Gender Smoking Diabetes	Traditional CVD	risk factors	
		Age	OR 1.05 (1.01–1.09)	
			OR 1.35 (2.78, 0.64)^a^	Being male
			OR 0.69 (0.23–2.07)	Smoker (yes v no)
		Diabetes	OR 2.38 (1.33–4.35) ^a^	
		Hypertension	OR 2.00 (NP) ^a^^,^^b^	
		Socioeconomic	Factors	
			OR 0.62 (NP)^b^	Working (no v yes)
			ref	Region: Al-Asimah
			OR 1.07 (NP)^b^	Hawalli
			OR 1.21 (NP)^b^	Al-Farwaniya
			OR 0.73 (NP)^b^	Mubarak Al-Kabir
		Psychological factors		
			OR 0.98 (NP)^b^	DASS Depression
			OR 0.96 (NP)^b^	DASS Anxiety
			OR 0.99 (NP)^b^	DASS Stress
		Treatment related	factors	
		Statin duration	OR 1.04 (NP)^b^	
			ref	Atorvastatin
			OR 0.29(NP)^b^	Rosuvastatin
			OR 1.64 (NP)^b^	Simvastatin
Braamskamp et al (2015)[32] Netherlands	All variables Entered into backward stepwise regression—adjusted effects not presented	Traditional CVD	related factors	
			OR 1.11(0.97–1.22)^b^	Age
			OR 1.28 (0.63–2.61)^b^	Male gender
			OR 0.90(0.70–1.19)^b^	LDL-C (pre-statin)
			OR 0.96(0.89–1.03)^b^	BMI
			OR 1.20 (0.58–2.46)^b^	CVD 1st degree relative
		Treatment related	factors	
			OR 1.39 (0.49–3.90)^b^	Use of concurrent meds
			OR 1.66 (0.77–3.58)^b^	Initiation statins < puberty
			OR 0.54 (0.31–1.87)^b^	Side effects
Harrison et al. (2013)[33] USA	NA	NA	NA	NA
**Retrospective**				
Aarnio et al. (2016)[34] Finland	Adjusted for all baseline characteristics: CVD risks, socioeconomic demographic, comorbidities, treatment related and cost related factors, and year of statin initiation	Traditional CVD	related factors	
		Gender (male v female)	OR 1.06 (1.03–1.09)^b^^,^^c^	
		**Male cohort**		
		Age (years): 45–49	Ref	
		50–54	OR 1.16 (1.10–1.23)^a^	
		55–59	OR 1.25 (1.18–1.33)^a^	
		60–64	OR 1.25 (1.18–1.35)^a^	^a^
		65–69	OR 1.27 (1.16–1.39)^a^	
		≥70	OR 1.20 (1.10–1.33))^a^	
		Diabetes mellitus	OR 1.14 (1.09–1.20)^a^	
		Hypertension	OR 1.09 (1.02–1.1.5)^a^	
			OR 1.05 (0.89–1.23)^a^	Dyslipidemia
		Num. of CV meds (per additional class)	OR 1.03 (1.00–1.06)^a^	
		Socioeconomic	Factors	
		Income (€/year): ≥31400	Ref	
		≤10200	OR 0.74 (0.68–0.79)^a^	
		10300–15300	OR 0.80 (0.75–0.85)^a^	
		15400–22000	OR 0.84 (0.79–0.89)^a^	
		22100–31300:	OR 0.94 (0.90–1.0)^a^	
		Education: Higher degree	Ref	
		Basic education	OR 0.92 (0.88–0.96)^a^	
		Secondary education	OR 0.91 (0.86–0.95)^a^	
		Labour status: Employed	Ref	
		Unemployed	OR 0.98 (0.92–1.05)^a^	
		Retired	OR 1.11 (1.05–1.19)^a^	
		Out of labour market	OR 0.90 (0.78–1.04)^a^	
		Marital Status: Single	Ref	
		Married	OR 0.85 (0.80-.91)^a^	
		Divorced	OR 0.61 (0.56-.67))^a^	
		Widowed	OR 0.79 (0.69-.92)^a^	
		Region: Southern	Ref	
		Southwestern	OR 0.90 (0.85–0.95)^a^	
		Central	OR 0.92 (0.88–0.96)^a^	
		Eastern	OR 0.98 (0.93–1.03)^a^	
		Northern	OR 0.83 (0.79–0.88)^a^	
		Co-morbidities:		
			OR 1.02 (0.97–1.06)^a^	CCI (per additional point)
			OR 1.14 (1.03–1.27)^a^	Atrial fibrillation
			OR 0.81 (0.64–1.02)^a^	Obesity
			OR 1.02 (0.90–1.16)^a^	Cancer
			OR 1.10 (0.93–1.28)^a^	Cardiac insufficiency
		COPD & asthma	OR 0.85 (0.79–0.91)^a^	
			OR 0.94 (0.83–1.08)^a^	Rheumatoid arthritis
			OR 1.27 (0.89–1.79)^a^	Renal Insufficiency
		Alcoholism/narcomania	OR 0.76 (0.63–0.92)^a^	
		Dementia	OR 2.17 (1.52–3.23)^a^	
		Depression	OR 0.85 (0.79–0.93)^a^	
		Mental Disorder	OR 1.41 (1.25–1.59)^a^	
		No. of hospital days: 0	Ref	
		1–4	OR 0.94 (0.90–0.98)	
		5–10	OR 0.93 (0.85–0.99)^a^	
			OR 0.93 (0.83–1.05)^a^	11–20
			OR 1.05 (0.90–1.22)^a^	≥21
		Use of NSAIDs	OR 0.88 (0.83–0.91)^a^	
			OR 0.99 (0.98–1.01)^a^	Per additional medicine
		Medication	Costs	
		Total out-of-pocket costs (per additional €50)	OR 1.12 (1.10–1.15)^a^	
		Co-payment dispensation (euro cents/tablet) <20	Ref.	
		20-<30	OR 0.90 (0.79–1.02)^a^	
		30-<60	OR 0.77 (0.70–0.85)^a^	
		60-<70	OR 0.73 (0.66–0.81)^a^	
		70-<90	OR 0.61 (0.55–0.68)^a^	
		90-<120	OR 0.53 (0.47–0.59)^a^	
		≥120	OR 0.38 (0.32–0.45)^a^	
		Treatment related	Factors	
		Type of statin: Simvastatin	Ref	
		Lovastatin	OR 0.84 (0.72–0.98)^a^	
			OR 0.99 (0.89–1.01)^a^	Pravastatin
		Fluvastatin	OR 1.12 (1.04–1.22)^a^	
		Atorvastatin	OR 1.30 (1.22–1.37)^a^	
		Rosuvastatin	OR 1.45 (1.33–1.59)^a^	
		Statin dose intensity: Low^1^	Ref	
		Moderate^2^	OR 0.89 (0.84–0.94)^a^	
		High^3^	OR 0.70 (0.54–0.92)^a^	
Aarnio et al. (2016)[34] Finland	Adjusted for all baseline characteristics: CVD risk, socioeconomic demographic, comorbidities, treatment related and cost related factors, and year of statin initiation	Traditional	CVD risk related	factors
		**Female cohort**		
		Age (years): 45–49	Ref	
		50–54	OR 1.08 (1.00–1.15)^a^	
		55–59	OR 1.22 (1.14–1.30)^a^	
		60–64	OR 1.22 (1.14–1.32)^a^	
		65–69	OR 1.23 (1.14–1.35)^a^	
		≥70	OR 1.27 (1.16–1.39)^a^	
		Diabetes mellitus	OR 1.19 (1.12–1.27)^a^	
			OR 1.03 (0.87–1.22)^a^	Dyslipidemia
			OR 1.01 (0.96–1.06)^a^	Hypertension
		Num. of CV Meds.(per additional class)	OR 1.04 (1.01–1.08)^a^	
		Socioeconomic	Factors	
		Income (€/year): ≥31400	Ref	
		≤10200	OR 0.93 (0.86–1.00)^a^	
		10300–15300	OR 0.95 (0.88–1.02)^a^	
		15400–22000	OR 1.00 (0.93–1.06)^a^	
		22100–31300:	OR 0.98 (0.93–1.05)^a^	
		Education: Higher degree	Ref	
		Basic education	OR 1.06 (1.01–1.11)^a^	
		Secondary education	OR 1.03 (0.98–1.09)^a^	
		Labour status: Employed	Ref	
		Unemployed	OR 1.06 (1.00–1.14)^a^	
		Retired	OR 1.11 (1.05–1.18)^a^	
		Out of labour market	OR 0.94 (0.84–1.05)^a^	
		Marital Status: Single	Ref	
		Married	OR 0.85 (0.79–0.90)^a^	
		Divorced	OR 0.68 (0.64–0.74)^a^	
		Widowed	OR 0.78 (0.72–0.85)^a^	
		Region: Southern	Ref	
		Southwestern	OR 0.93 (0.88–0.97)^a^	
		Central	OR 1.00 (0.96–1.04)^a^	
		Eastern	OR 1.08 (1.03–1.12)^a^	
		Northern	OR 0.90 (0.85–0.95)^a^	
		Co-morbidities:		
			OR 1.01 (0.96–1.05)^a^	CCI (per additional point)
		Obesity	OR 0.76 (0.61–0.93)^a^	
		Atrial fibrillation	OR 0.90 (0.80–1.02)^a^	
			OR 0.99 (0.84–1.17)^a^	Cardiac insufficiency
		Alcoholism/narcomania	OR 0.53 (0.41–0.69)^a^	
		Dementia	OR 1.41 (1.10–1.82)^a^	
		Depression	OR 0.91 (0.85–0.95)^a^	
		Mental Disorder	OR 1.35 (1.23–1.49)^a^	
		Cancer	OR 1.11 (1.00–1.23)^a^	
		COPD & asthma	OR 0.82 (0.78–0.86)^a^	
			OR 1.41 (0.96–2.04)^a^	Renal Insufficiency
		Rheumatoid arthritis	OR 0.90 (0.82–0.99)^a^	
		No. of hospital days: 0	Ref	
		1–4	OR 0.93 (0.89–0.96)^a^	
		5–10	OR 0.91 (0.85–0.97)^a^	
		11–20	OR 0.90 (0.81–1.00)^a^	
		≥21	OR 1.02 (0.89–1.16)^a^	
		Use of NSAIDs	OR 0.92 (0.88–0.95)^a^	
		Hormone therapy	OR 1.09 (1.05–1.12)^a^	
			OR 0.99 (0.98–1.01)^a^	Per additional medicine
		Cost related	Factors	
		Total out-of-pocket costs (per additional €50)	OR 1.12 (1.11–1.15)^a^	
		Co-payment dispensation (euro cents/tablet) <20	Ref	
		20-<30	OR 0.75 (0.67–0.83)^a^	
		30-<60	OR 0.68 (0.63–0.68)^a^	
		60-<70	OR 0.68 (0.63–0.68)^a^	
		70-<90	OR 0.57 (0.52–0.63)^a^	
		90-<120	OR 0.48 (0.43–0.53)^a^	
		≥120	OR 0.37 (0.32–0.45)^a^	
		Treatment related	Factors	
		Type of statin: Simvastatin	Ref	
		Lovastatin	OR 0.86 (0.76–0.97)^a^	
		Pravastatin	OR 0.99 (0.90–1.08)^a^	
		Fluvastatin	OR 1.15 (1.11–1.22)^a^	
		Atorvastatin	OR 1.16 (1.11–1.22)^a^	
		Rosuvastatin	OR 1.32 (1.23–1.43)^a^	
		Statin dose intensity: Low^1^	Ref	
		Moderate^2^	OR 0.90 (0.85–0.85)^a^	
		High^3^	OR 0.61 (0.45–0.82)^a^	
Batal et al. (2007)[35] USA	Gender Age Race/ethnicity Insurance status Co-payment Number of comorbidities	Traditional	CVD risk related	factors
		Male gender	RR 1.09 (1.02–1.16)^a^	
		Age (per 10 yr increase)	RR 1.07 (1.03–1.10)^a^	
			OR 0.90 (0.79–1.04)	Use of diabetics
		Co-morbidities:		
		Num. of Comorbidities	RR 1.04 (1.03–1.06)^a^	
		Demographic factors:		
		Race: Whited	Ref	
		Black	RR .77 (0.70–0.86)^a^	
		Hispanic	RR .77 (0.70–0.86)^a^	
			RR 1.02 (0.91–1.16)^a^	Other
		Treatment related	Factors	
		60 versus 30 day supply	RR 1.40 (1.27–1.55)^a^	
		Cost related	Factors	
			OR 1.09 (0.94–1.26)	Insurance
			OR 1.0 (0.92–1.24)	Co-payment
Corrao et al. (2010)[37] Italy	None	Traditional	CVD risk related	factors
		Male gender	OR 1.23 (1.19–1.27)^b^^,^^c^	
		Age (years)	MD 1.7 (1.50–1.90)^c^^,^^d^	
		Antidiabetics (yes)	OR 0.26 (0.24–0.29)^b^^,^^c^	
		Co-morbidities:		
		CCI score = 0	Ref	
		1	OR 2.3 (2.0–2.6) ^c^^,^^d^	
		2	OR 2.30 (2.17–2.44)^b^^,^^c^	
		Antihypertensives	OR 0.07 (0.07–0.08)^b^^,^^c^	
		Digitalis or organic nitrates	OR 0.25 (0.24–0.27)^b^^,^^c^	
		Other cardiac drugs	OR 0.23 (0.24–0.25)^b^^,^^c^	
		Treatment related	Factors	
		Type of statin: Simvastatin	Ref	
			OR 1.02 (0.97–1.08) ^b^^,^^c^	Pravastatin
		Fluvastatin	OR 2.3 (2.17–2.44) ^b^^,^^c^	
		Atorvastatin	OR 2.58 (2.45–2.71) ^b^^,^^c^	
		Statin switching (yes)	OR 0.52 (0.47–0.57) ^b^^,^^c^	
Bryson et al. (2008)[36] USA	Age Gender Marital status Race/Ethinicity Education Number of Medications Smoking status Depression	Alcohol misuse: None	Ref	
			OR 0.95 (0.82–1.10)^d^	Low drinker
			OR 1.03 (0.83–1.27)^d^	Mild misuse
			OR 1.00 (0.72–1.38)^d^	Moderate misuse
		Severe misuse	OR 0.68 (0.48–0.96)^d^	
		Alcohol misuse: None	Ref	
			OR 0.99 (0.90–1.09)^d^	Low drinker
			OR 0.99 (0.86–1.14)^d^	Mild misuse
			OR 1.00 (0.81–1.24)^d^	Moderate misuse
		Severe misuse	OR 0.73 (0.56–0.96)^d^	
Halava et al. (2015)[38] Sweden	Time, calendar year, age at retirement, primary prevention*time	Socioeconomic	Factors	
		Retirement (adj. for age)	PR 0.85(0.80–0.88)^d^	
		Retirement (adj. for age)	PR 0.85(0.81–0.90)^d^	
Lavikainen et al. (2016)[39] Finland	None	Traditional	CVD risk related	factors
		Age 45–49 years	Ref	
		50–54 years	OR 1.11 (1.03–1.19)^b^^,^^c^^,^^e^	
		55–59 years	OR 1.30 (1.22–1.39) ^b^^,^^c^^,^^e^	
		60–64 years	OR 1.33 (1.24–1.42) ^b^^,^^c^^,^^e^	
			OR 0.87 (0.72–1.05) ^b^^,^^c^^,^^e^	Dyslipidemia
		Diabetes (yes v no)	OR 1.21 (1.14–1.29) ^b^^,^^c^^,^^e^	
		Use of insulin (yes v no)	OR 1.13 (1.01–1.25) ^b^^,^^c^^,^^e^	
		Hypertension	OR 1.13 (1.09–1.18) ^b^^,^^c^^,^^e^	
			OR 1.00 (0.77–1.32) ^b^^,^^c^^,^^e^	Heart failure
		Number of CVD meds- 0	Ref	
		1	OR 1.18 (1.13–1.23) ^b^^,^^c^^,^^e^	
		2	OR 1.19 (1.12–1.25) ^b^^,^^c^^,^^e^	
		3–6	OR 1.21 (1.11–1.31) ^b^^,^^c^^,^^e^	
		Socioeconomic	Factors	
			Ref	Income (€) ≤11,200
			OR 1.01 (0.96–1.07) ^b^^,^^c^^,^^e^	11,300–18,700
			OR 0.99 (0.94–1.05) ^b^^,^^c^^,^^e^	18,800–25,400
			OR 1.00 (0.95–1.06) ^b^^,^^c^^,^^e^	≥25,500
		Region: Helsinki	Ref	
		Turku	OR 0.93 (0.88–0.99) ^b^^,^^c^^,^^e^	
			OR 1.01 (0.95–1.06) ^b^^,^^c^^,^^e^	Tampere
		Kuopio	OR 1.06 (1.0–1.11) ^b^^,^^c^^,^^e^	
		Oulo	OR 0.82 (0.77–0.87) ^b^^,^^c^^,^^e^	
		Education: Higher degree	Ref	
		Basic level	OR 1.05 (1.00–1.11)^b^^,^^c^^,^^e^	
			OR 0.98 (0.93–1.03) ^b^^,^^c^^,^^e^	Secondary level
		Marital status-married	Ref	
		Divorced	OR 0.82 (0.78–0.86) ^b^^,^^c^^,^^e^	
		Unmarried	OR 1.10 (1.03–1.17) ^b^^,^^c^^,^^e^	
		Labour status- employed	Ref	
		Unemployed	OR 1.05 (1.00–1.11) ^b^^,^^c^^,^^e^	
		Retired	OR 1.16 (1.11–1.21) ^b^^,^^c^^,^^e^	
			OR 0.95 (0.86–1.04) ^b^^,^^c^^,^^e^	Out of labour market
		Comorbidities°		
		CCI≥1	OR 1.08 (1.00–1.16) ^b^^,^^c^	
		Cancer	OR 1.15 (1.04–1.27)^b^^,^^c^^,^^e^	
			OR 0.96 (0.85–1.09) ^b^^,^^c^^,^^e^	Cardiac arrhythmia
			OR 1.05 (0.99–1.11) ^b^^,^^c^^,^^e^	Respiratory diseases
			OR 1.02 (0.90–1.14) ^b^^,^^c^^,^^e^	Rheumatoid Arthritis
		Alcohol-related diseases	OR 0.62 (0.48–0.81) ^b^^,^^c^^,^^e^	
			OR 1.05 (0.99–1.11) ^b^^,^^c^^,^^e^	Depression
		Mental Disorders	OR 1.36 (1.21–1.53 ^b^^,^^c^^,^^e^	
			OR 1.01 (0.88–1.15) ^b^^,^^c^	Anxiolytics, hypnotics
			0.90 (0.85–1.03) ^b^^,^^c^	Corticosteroids
		NSAID use	OR 0.96 (0.93–1.00) ^b^^,^^c^^,^^e^	
		Hormone therapy	OR 1.15 (1.11–1.20) ^b^^,^^c^^,^^e^	
		Number of meds.		
		1–2	Ref	
		3–5	OR 1.08 (1.04–1.14) ^b^^,^^c^	
		6–31	OR 1.23 (1.17–1.29) ^b^^,^^c^	
		Number of in-hospital days– 0	Ref	
		1–2	OR 0.90 (0.86–0.95) ^b^^,^^c^^,^^e^	
		3–6	OR 0.91 (0.86–0.97) ^b^^,^^c^^,^^e^	
			OR 0.97 (0.89–1.04) ^b^^,^^c^^,^^e^	8–321
		Treatment related	Factors	
		Type of statin:		
		Simvastatin	Ref	
		Lovastatin	OR 0.80 (0.69–0.93) ^b^^,^^c^^,^^e^	
		Pravastatin	OR 0.65 (0.60–0.71) ^b^^,^^c^^,^^e^	
		Fluvastatin	OR 1.09 (1.01–1.17) ^b^^,^^c^^,^^e^	
		Rosuvastatin	OR 1.53 (1.37–1.80) ^b^^,^^c^^,^^e^	
			OR 1.01 (0.96–1.05) ^b^^,^^c^^,^^e^	Atorvastatin
		Year statin initiated-2001	Ref	
		2002	OR 1.06 (1.00–1.12) ^b^^,^^c^	
		2003	OR 1.17 (1.10–1.23) ^b^^,^^c^	
		2004	OR 1.34 (1.27–1.41) ^b^^,^^c^	
		Stain dosing- Low^a^	Ref	
		Moderate^b^	OR 0.92 (0.89–0.96) ^b^^,^^c^^,^^e^	
		High^c^	OR 0.47 (0.34–0.64) ^b^^,^^c^^,^^e^	
Perrault et al. (2009)[40] Canada	None	Traditional	CVD risk related	factors
		Age (years)	MD 0.5 (0.42–0.58)^b^^,^^f^	
		Male gender	OR 0.96 (0.94–0.97) ^b^^,^^c^	
		Hypertension	OR 1.59 (1.56–1.62) ^b^^,^^c^	
		Diabetes	OR 1.39 (1.36–1.42) ^b^^,^^c^	
		Socioeconomic	Factors	
		Social assistance	OR 1.17 (1.38–1.47) ^b^^,^^c^	
		Comorbidities		
		Chronic disease score (≥4)	OR 1.43 (1.14–1.20)^b^^,^^c^	
		Respiratory disease	OR 1.05 (1.01–1.08) ^b^^,^^c^	
		Use of antidepressants	OR 1.21 (1.17–1.24) ^b^^,^^c^	
		Use of anxiolytics0.25	OR 1.20 (1.17–1.24) ^b^^,^^c^	
Perrault et al. (2009a)[41] Canada	None	Traditional	CVD risk related	factors
		Age (years)	MD 1.0 (0.84–1.16)^b^^,^^f^^,^^g^	
		Male gender	OR 0.96 (0.93–0.99) ^b^^,^^c^^,^^g^	
		Hypertension	OR 1.35 (1.31–1.40) ^b^^,^^c^^,^^g^	
		Diabetes	OR 1.31 (1.27–1.36) ^b^^,^^c^^,^^g^	
		Socioeconomic	Factors	
		Social assistance	OR 1.15 (1.10–1.20) ^b^^,^^c^^,^^g^	
		Comorbidities		
		Chronic disease score ≥4	OR 1.09 (1.05–1.14) ^b^^,^^c^^,^^g^	
		Use of antiplatelets	OR 1.23 (1.18–1.30) ^b^^,^^c^^,^^g^	
		Treatment related	Factors	
		Type of statin:		
		Simvastatin	Ref	
		Lovastatin	OR 7.59 (6.71–8.58) ^b^^,^^c^	
		Pravastatin	OR 0.05 (0.05–0.06) ^b^^,^^c^	
		Fluvastatin	OR 0.72 (0.64–0.81) ^b^^,^^c^	
		Rosuvastatin	OR 0.68 (0.59–0.78) ^b^^,^^c^	
			OR 0.92 (0.83–1.03) ^b^^,^^c^	Atorvastatin
Rublee et al. (2012)[42] USA	None	Traditional	CVD risk related	factors
		Age (years)	MD 2.3 (2.19–2.41)^b^^,^^f^	
		Male gender	OR 1.04 (1.02–1.08) ^b^^,^^c^	
		Hypertension	OR 1.21 (1.18–1.25) ^b^^,^^c^	
		Diabetes	OR 0.86 (0.84–0.89) ^b^^,^^c^	
		Use of beta blockers	OR 1.34 (1.29–1.39) ^b^^,^^c^	
		Use of ACE inhibitors	OR 1.37 (1.32–1.41) ^b^^,^^c^	
		Use of ARBs	OR 1.24 (1.18–1.30) ^b^^,^^c^	
		Use of Diuretics	OR 1.27 (1.23–1.32) ^b^^,^^c^	
		Use of anticoagulants	OR 1.41 (1.29–1.55) ^b^^,^^c^	
		Use of antiplatelet agents	OR 1.05 (0.95–1.15) ^b^^,^^c^	
		Use of vasodilators	OR 1.11 (0.96–1.28) ^b^^,^^c^	
		Use of digitalis	OR 3.12 (2.67–3.63) ^b^^,^^c^	
		Socioeconomic	Factors	
		Region: Midwest	Ref	
		Northeast	OR 0.87 (0.83–0.91) ^b^^,^^c^	
		Southeast	OR 0.67 (0.65–0.70) ^b^^,^^c^	
		South	OR 0.68 (0.65–0.70) ^b^^,^^c^	
		West	OR 1.08 (1.03–1.13) ^b^^,^^c^	
		Comorbidities		
		CCI = 0	Ref	
		1	OR 1.06 (1.01–1.18) ^b^^,^^c^	
		2	OR 1.09 (1.05–1.13) ^b^^,^^c^	
		3	OR 0.75 (0.70–0.80) ^b^^,^^c^	
			OR 0.98 (0.92–1.04) ^b^^,^^c^	≥4
		Obesity	OR 0.83 (0.78–0.88) ^b^^,^^c^	
			OR 1.01 (0.96–1.06) ^b^^,^^c^	Depression
		COPD	OR 1.21 (1.12–1.31) ^b^^,^^c^	
		Dementia	OR 2.00 (1.36–2.94) ^b^^,^^c^	
		Chronic Kidney Disease	OR 1.14 (0.97–1.35) ^b^^,^^c^	
		Cancer	OR 1.34 (1.26–1.43) ^b^^,^^c^	
		Medication	Beliefs/behaviours	
		General physical exam	OR 1.20 (1.17–1.23) ^b^^,^^c^	
		Bone mineral density test	OR 1.36 (1.28–1.44) ^b^^,^^c^	
		Screening Mammography	OR 1.53 (1.47–1.60) ^b^^,^^c^	
		Papanicolaou test	OR 1.16 (1.11–1.21) ^b^^,^^c^	
		PSA testing	OR 1.17 (1.13–1.22) ^b^^,^^c^	
		Fecal occult blood tests	OR 1.09 (1.05–1.13) ^b^^,^^c^	
		Influenza vaccinations	OR 1.31 (1.26–1.36) ^b^^,^^c^	
		Pneumococcal vacc.	OR 1.31 (1.19–1.43) ^b^^,^^c^	
		Cost related	Factors	
		Health plan type:		
		Point of service	Ref	
		Preferred provider	OR 1.09 (1.00–1.20) ^b^^,^^c^	
			OR 1.00 (0.97–1.03) ^b^^,^^c^	Health maintenance
		Exclusive provider	OR 0.51 (0.48–0.54) ^b^^,^^c^	
		Indemnity	OR 2.05 (1.89–2.23) ^b^^,^^c^	
		Other	OR 0.68 (0.62–0.75) ^b^^,^^c^	
Slejko et al. (2014)[43] USA	None	Traditional	CVD risk related	factors
			OR 1.14 (1.05–1.24) ^b^^,^^c^	Gender
		Age: over 65 years old	OR 1.19 (1.03–1.37) ^b^^,^^c^	
			OR 1.03 (0.91–1.17) ^b^^,^^c^	History of diabetes
		History of hypertension	OR 1.16 (1.04–1.28)	
		Socioeconomic	Factors	
		Region: Midwest	Ref	
		Northeast	OR 0.82 (0.72–0.93) ^b^^,^^c^	
		South	OR 0.68 (0.59–0.79) ^b^^,^^c^	
		West	OR 0.79 (0.65–0.95) ^b^^,^^c^	
		Treatment related	Factors	
		Prescribing Physician: General practitioner	Ref	
			OR 0.92 (0.80–1.06) ^b^^,^^c^	Internist
		Cardiologist	OR 3.91 (3.07–4.98) ^b^^,^^c^	
			OR 0.93 (0.83–1.06) ^b^^,^^c^	Other/unknown
		Cost related	Factors	
			Ref	Plan type: Commercial
			OR 0.84 (0.67–1.05) ^b^^,^^c^	Medicare
			OR 1.07 (0.44–2.58) ^b^^,^^c^	Medicaid
			OR 1.46 (0.1–1.2) ^b^^,^^c^	Other
Wallach-Kildemoes et al. (2014)[44] Denmark	Age Income Education Hypertension	Men aged 40–64 years		
		Traditional	CVD risk related	factors
		Age (years): 40–44	Ref	
		45–49	OR 1.16 (1.16–1.16)^a^	
		50–54	OR 1.43 (1.43–1.43)^a^	
		55–59	OR 1.54 (1.54–1.54)^a^	
		60–64	OR 1.85 (1.85–1.89)^a^	
		Hypertension	OR 1.43 (1.41–1.43)^a^	
		Socioeconomic	Factors	
		Income: 1. Lowest	Ref	
		2	OR 1.27 (1.27–1.27)^a^	
		3	OR 1.41 (1.41–1.41)^a^	
		4	OR 1.59 (1.56–1.59)^a^	
		5. Highest	OR 1.56 (1.54–1.56)^a^	
		Education (years): 7–10	Ref	
			OR 1.00 (1.00–1.00)^a^	10–12
		≥ 12	OR 1.03 (1.03–1.03)^a^	
	Age Income Education Hypertension	Women aged 40–64 years		
		Traditional	CVD risk related	factors
		Age (years): 40–44	Ref	
		45–49	OR 1.33 (1.32–1.33)^a^	
		50–54	OR 1.61 (1.61–1.64)^a^	
		55–59	OR 1.82 (1.82–1.85)^a^	
		60–64	OR 1.96 (1.96–1.96)^a^	
		Hypertension	OR 1.33(1.33–1.33)^a^	
		Socioeconomic	Factors	
		Income: 1. Lowest	Ref	
		2	OR 1.16 (1.16–1.16)^a^	
		3	OR 1.30 (1.28–1.30)^a^	
		4	OR 1.32 (1.32–1.32)^a^	
		5. Highest	OR 1.27 (1.27–1.27)^a^	
		Education (years): 7–10	Ref	
		10–12	OR 0.90 (0.90–0.90)^a^	
		≥ 12	OR 0.85 (0.85–0.85)^a^	
	Age Income Education Hypertension	Men aged 65–84 years		
		Traditional	CVD risk related	factors
		Age (years): 65–79	Ref	
		70–75	OR 1.01 (1.01–1.01)^a^	
		75–79	OR 0.92 (0.92–0.93)^a^	
		80–84	OR 0.63 (0.63–0.63)^a^	
		Hypertension	OR 1.47 (1.47–1.47)^a^	
		Socioeconomic	Factors	
		Income: 1. Lowest	Ref	
		2	OR 1.22 (1.22–1.22)^a^	
		3	OR 1.22 (1.22–1.22)^a^	
		4	OR 1.30 (1.30–1.30)^a^	
		5. Highest	OR 1.37 (1.37–1.37)^a^	
		Education (years): 7–10	Ref	
		10–12	OR 0.99 (0.99–0.99)^a^	
		≥ 12	OR 1.03 (1.03–1.03)^a^	
	Age Income Education Hypertension	Women aged 65–84 years		
		Traditional	CVD risk related	factors
		Age (years): 65–79	Ref	
		70–75	OR 0.86 (0.85–0.86)^a^	
		75–79	OR 0.65 (0.65–0.65)^a^	
		80–84	OR 0.61 (0.61–0.62)^a^	
		Hypertension	OR 1.27 (1.27–1.27)^a^	
		Socioeconomic	Factors	
		Income 1. Lowest	Ref	
		2	OR 1.14 (1.14–1.14)^a^	
		3	OR 1.09 (1.08–1.09)^a^	
		4	OR 1.09 (1.09–1.09)^a^	
		5. Highest	OR 1.05 (1.05–1.06)^a^	
		Education (years): 7–10	Ref	
		10–12	OR 0.91 (0.90–0.91)^a^	
		≥ 12	OR 0.91 (0.90–0.91)^a^	
**Prospective**	**cohort studies**			
Farsaei et al. (2015)[45] Iran	None	Traditional	CVD risk related	factors
			OR 1.37 (0.58–3.22)	Female gender
			MD 2.6 (-0.47–5.67)^b^^,^^f^	Age (years)
		Socioeconomic	Factors	
				Education level:
			Ref	Illiterate
			OR 0.96 (0.35–2.67)^b^^,^^c^	Primary
			OR 0.84 (0.26–2.70) ^b^^,^^c^	Secondary
			OR 0.99 (0.25–3.95) ^b^^,^^c^	Degree or higher
		Comorbidities		
		Num. of medications	MD 1.4 (0.98–1.82)^b^^,^^f^	
		Lifestyle	Factors	
			MD -0.1 (-.1.69–1.49)^b^^,^^f^	BMI
Halava et al. (2014)[46] Finland	Gender, age Education, region of birth, Marital status, Cancer Depression, Self-rated health	Traditional	CVD risk related	factors
		Smoking -None	Ref	
		Ex-smoker	OR 1.20 (1.0–1.3)^a^	
			OR 1.01 (0.86–1.18)^a^	Current smoker
		Lifestyle	factors	
		BMI <25	Ref	
		BMI 25–29.9	OR 0.88 (0.79–0.98)^a^	
		BMI ≥30	OR 0.86 (0.74–0.99)^a^	
			Ref	Alcohol use: None
			OR 0.92 (0.79–1.06)^a^	Moderate
			OR 0.88 (0.70–1.11)^a^	High
			OR 0.99 (0.71–1.23)^a^	Extreme drinking (yes)
			Ref	Physical activity: Low
			OR 0.99 (0.87–1.12)^a^^,^^b^^,^^c^	Moderate
			OR 1.00 (0.89–1.13) ^a^^,^^b^^,^^c^	Active
			Ref	Num. of risks: 0
			OR 0.93 (0.85–1.04)^a^	1–2
			OR 1.15 (1.52–0.87)^a^	3–4
Mann et al. (2007)[47] USA	None	Traditional	CVD risk related	factors
		Age ≥50 years	OR 6.65 (1.16–37.88) ^b^^,^^c^	
			OR 1.45 (0.44–4.78) ^b^^,^^c^	Treated for hypertension
		Socioeconomic	Factors	
			OR 0.30 (0.06–1.58) ^b^^,^^c^	Race–Hispanic
			OR 0.34 (0.06–1.87) ^b^^,^^c^	Some college
		Comorbidities		
			OR 1.76 (0.42–7.34) ^b^^,^^c^	Has comorbidity
		Medication	Beliefs/behaviours	
		Had cholesterol check	OR 4.75(1.17–19.24) ^b^^,^^c^	
			OR 3.31 (0.73–13.76) ^b^^,^^c^	Taking BP pills
			OR 0.34 (0.08–1.43) ^b^^,^^c^	Learnt more diet changes
		Risk of MI < average	OR 0.15 (0.04–0.61) ^b^^,^^c^	
			OR 0.94 (0.05–2.18) ^b^^,^^c^	Do not worry about chol.
			OR 4.51 (0.80–21.82) ^b^^,^^c^	Pills cure high chol.
			OR 0.20 (0.04–1.08) ^b^^,^^c^	Will take pill rest of life
		Do not expect to take statin rest of life	OR 0.20 (0.05–0.86) ^b^^,^^c^	
			OR 0.94 (0.24–3.67) ^b^^,^^c^	Do not need pill
			OR 0.65 (0.15–3.67) ^b^^,^^c^	Taking pill same or harder than diet control
			OR 0.89 (0.25–3.10) ^b^^,^^c^	Have concerns (statins)
			OR 0.31 (0.07–1.31) ^b^^,^^c^	The pill may be harmful
	All variables in univariate analyses with *p*<0.2 entered into stepwise regression	Traditional	CVD risk related	factors
		Age ≥50 years	OR 4.2 (1.1–15.8) ^a^	
		Socioeconomic	Factors	
		Race–Hispanic	OR 0.26 (0.07–1.0) ^a^	
		Medication	Beliefs/behaviours	
		Plan to use statins <6 mo.	OR 0.28 (0.11–0.71)^a^	
		Risk of MI < average	OR 0.32 (0.11–0.91) ^a^	
		Statin may be harmful	OR 0.40 (0.16–1.0) ^a^	
RCT				
Guthrie (2001)[48] USA	None	Medication	Beliefs/behaviours	
		Seeing physician	OR 1.25 (1.07–1.45) ^b^^,^^c^	
		Changed eating habits	OR 1.59 (1.35–1.88) ^b^^,^^c^	
			OR 1.18 (0.99–1.39) ^b^^,^^c^	Lost weight
		Increased physical activity	OR 1.53 (1.28–1.82) ^b^^,^^c^	
			OR 1.25 (0.97–1.62) ^b^^,^^c^	Tried to quit smoking
		Improved BP control	OR 1.43 (1.21–1.70) ^b^^,^^c^	
			OR 1.19 (0.89–1.58) ^b^^,^^c^	Improved diabetes control
Stilley et al. (2004)[49] USA	Psychological distress, IQ Attention, Concs. Mental Flexibility/ Perceptual organization.	Conscientiousness (Concs.)	*B* .24 (NP)^b^	
		Anxiety	*B*-0.16 (NP)^b^	
		Depression	*B*-0.24 (NP)^b^	
		Estimated IQ	*B* 0.25 (NP)^b^	
		Attention	*B*-0.16 (NP)^b^	
		Mental Flexibility	*B*-0.21 (NP)^b^	
		Visuospatial/ construction	*B*-0.21 (NP)^b^	
			*B*-0.05 (NP)^b^	Neuroticism
			*B* 0.03 (NP)^b^	Extroversion
			*B*-0.08 (NP)^b^	Openness
			*B* 0.03 (NP)^b^	Agreeableness
			*B* 0.06 (NP)^b^	Verbal learning
			*B* 0.03 (NP)^b^	Verbal Recall
			*B* 0.03 (NP)^b^	Nonverbal memory
		Conscientiousness	*B* 0.47 (NP)	
		Estimated IQ	*B* 0.22 (NP)	
			*B* -0.05 (NP)	Psychological distress
			*B* 0.07 (NP)	IQ*Conscientiousness

SEP: Socioeconomic position; CCI: Charlson Comorbidity Index;

^a^Effect size inverted to predict adherence;

^b^Unadjusted analyses;

^c^calculated from proportions;

^d^calculated from proportion estimates;

^e^predictors from a subsample of Aarnio et al.;

^f^calculated from means

^g^predictor from a subsample of Perrault 2009; MMAS-8; Morisky Medication Adherence Scale; DASS; Depression, Anxiety and Stress Scale; MASRI VAS; Medication Adherence Self-Report Inventory Visual Analogue Scale; MARS: Medication Adherence Report Scale; MAS: Morisky Adherence Scale;

^1^Fluvastatin 20–40mg, lovastatin 20mg, pravastatin 10–20mg, simvastatin 5–10mg;

^2^Atorvastatin 10–20mg, fluvastatin 80mg, lovastatin 40mg, pravastatin 40mg, rosuvastatin 10mg, simvastatin 20–40mg;

^3^Atorvastatin 40–80mg, rosuvastatin 20–40mg, simvastatin 60-80mg.

### Other demographic factors

Three studies provided data on other demographic factors [34,35,47].

#### Being married negatively associates with statin adherence—Strength of evidence = 3

Aarnio et al. reported that, compared to single men, married men after adjustment for other covariates were less likely to adhere to statins (OR 0.85, 955CI;0.80–0.91), as were divorced men (OR 0.61 95%CI; 0.56–0.67) and widowed men (OR 0.79, 95%CI; 0.69–0.79). The effects were very similar in the cohort of women [34]. The finding that married men and women are less adherent than single men and women is surprising, since married individuals generally benefit from spousal support that should ease not hinder adherent behaviour, therefore further research is required.

#### Racial background associates with adherence—Strength of evidence = 3

The effect of race on statin adherence was investigated in two US studies. In adjusted analyses Mann et al. reported being Hispanic American reduced the odds of adherence compared to being White American (OR 0.26, 95%CI;0.07–1.0) [47]. Batal et al reported the relative risk of being Hispanic American on statin adherence and, after adjustment for demographic and clinical factors and treatment costs, being Hispanic reduced the likelihood of adherence (RR 0.77, 95%CI; 0.72–0.84). Batal et al. also reported the likelihood of Black Americans adhering compared to White Americans was lower (RR 0.77, 95%CI; 0.70–0.86) [35]. These findings can be interpreted within the context of previous findings that in the US Black and Hispanic Americans face more barriers to adhering to statins such as lower levels of insurance and access to care. Given the increased prevalence of CVD risk factors such as hypertension and diabetes in Black and Hispanic US populations, this finding is particularly of note.

### Comorbid conditions

It was possible to investigate the association between co-morbidity and adherence on ten studies [34,35,37,39–42,45,47,49]. Given the increasing likelihood of comorbid conditions as people age and the positive association between age and adherence the unadjusted analyses should be interpreted cautiously.

#### The effect of increasing comorbidity on statin adherence: Strength of evidence = 0

The number of co-morbid conditions as a measure of disease burden was counted in six studies [34,35,39,40,42,47]. One large high quality study reported the odds of being adherent increased per additional comorbidity, after adjustment for age, gender, ethnicity and co-payment status [35]. One small high quality study reported that any co-morbidity increased the odds of self-reported adherence by a factor of ten, but this was unadjusted for other factors [47]. Three of these seven studies [34,39,42] calculated the Charlson Comorbidity index (CCI) [50], a validated measure of disease burden. Unadjusted effects from Lavikainen et al. reveal that participants with a CCI≥1 were more likely to be adherent than those with no comorbidity (OR 1.08 95%CI;1.00–1.16)) [39]. Similar sized unadjusted effects of having a score of one or two on the CCI compared to zero were reported by Rublee et al. [42]; however adherence was lower in the group with a CCI ≥3 compared to zero comorbidities (OR 0.75, 95%CI; 0.70–0.80). In contrast, Aarnio et al. who used the same registry data as Lavikainen et al. found no association between a one point increase in the CCI and the odds of being adherent in either men or women after adjusting for the other covariates [34]. Perrault et al. used a different measure of comorbidity, the chronic disease score [51], and people classed as having a high chronic disease score (≥4) were more adherent (OR 1.43, 95%CI;1.14–1.20) [39].

#### Depression inversely associates with statin adherence—Strength of evidence = 3

The existing evidence from five studies suggests that a diagnosis of depression does impact statin adherence [31,34,40,42,49]. In one study (n = 116846, QA score = 16) depression (identified from ICD-10 codes in Finnish registers) inversely associated with good adherence after adjustment for other covariates including age and socioeconomic factors in men (OR 0.85, 95%CI; 0.79–0.93) and in women (OR 0.91, 95%CI; 0.85–0.95) [34]. One low quality study (n = 158, QA score = 10) found an inverse association between depression and statin adherence using the Hamilton Depression Rating Scale (HDRS), where higher scores indicate increasingly depressive symptomatology [52], and adherence was measured using a Medication Event Monitoring System (MEMS) [49]. Unadjusted analyses from one large low quality study that identified depression from ICD-9 classification codes found no association between depression and adherence, nor did one small low quality study that used the Depression Anxiety and Stress Scale to measure depression [31,42]. Finally, data from one low quality study included use of antidepressants and this associated positively with adherence (OR 1.21, 95%CI; 1.17–1.24).

#### Association of anxiety with statin adherence—strength of evidence = 0

Four studies captured anxiety data and unadjusted effects were calculated [31,39,40,49]. Two studies found (n = 42046, n high quality = 0) that anxiety did not associate with adherence [30,38]. Two studies found anxiety did associate with adherence (n = 243072, n high quality = 0) [40,49]. Currently, the extent to which anxiety associates with adherence is poorly understood and evidenced.

#### The association of other mental health diagnoses and statin adherence—strength of evidence = 3

Aarnio et al found the presence of a “mental disorder” increased the odds of being adherent to statins by approximately 40% in men and women after adjustment for other comorbid conditions, socioeconomic, demographic and clinical factors [34]. Mental disorder here refers to the ICD10 codes for schizophrenia, psychotic, bipolar and manic disorders; there is evidence that cardiovascular mortality is higher in these groups and people may be informed of their increased risk and therefore adhere accordingly.

#### Obesity inversely associated with statin adherence—Strength of evidence = 3

Aarnio et al. found that obesity was associated with a lower odds of being adherent in women by about 25%, the same size of effect was observed in men but the confidence intervals crossed one (OR 0.81, 95%CI; 0.64–1.02) [33]. Rublee et al reported a very similar size of negative effect (OR 0.83, 95%CI; 0.78–0.88) [42]. Three studies collected data on BMI; Halava et al (n = 6458, QA score = 16) found, after adjustment for other clinical (depression, cancer and self-rated health), demographic and lifestyle factors, people classified as being obese (BMI>29.9) or overweight (25<BM<29.9 kg/m2) were approximately 15% less likely to be adherent than people with a BMI≤25 kg/m^2^ [46]. Importantly, Halava et al. considered cardiovascular comorbidities and risks for CVD including diabetes and hypertension to moderate the relationship between lifestyle and adherence and these people were excluded from this particular analysis. In contrast, two studies (n = 327, n high quality = 0) found that BMI did not differ across adherent and non-adherent groups [31,45].

#### Other co-morbid conditions: Strength of evidence = 3

Aarnio et al. and Rublee et al. included data on other comorbid conditions, these were identified from ICD10 and ICD9 codes collected in their respective Finnish and US registers (n = 195856, n high quality = 1) [34,42]. Aarnio et al. (QA score = 16) calculated adjusted odds ratios for men and women separately [33], whereas data provided in the Rublee study (QA score = 13) were used to calculate univariate odds ratios without stratification by gender [41].

Data were collected on cancer, respiratory disease, renal disease and rheumatoid arthritis. A diagnosis of cancer associated with good statin adherence in the US cohort. (OR 1.34, 95%CI; 1.26–1.43), Aarnio et al reported a smaller positive effect of cancer with stain adherence in women (OR 1.11, 95%CI; 1.00–1.23) but not men (OR 1.02, 95%CI; 0.90–1.16) [33]. A diagnosis of asthma/chronic obstructive pulmonary disease (COPD) reduced the odds of adherence for both men and women (OR 0.85, 95%CI; 0.79–0.91) in Aarnio et al., whereas in the US registry study Rublee et al. found a diagnosis of COPD increased the likelihood of adherence to statins (OR 1.21, 95%CI; 1.12–1.31). Neither Aarnio et al. nor Rublee et al. reported an association between renal insufficiency and adherence in (OR 1.27, 95%CI; 0.89–1.79) or women (OR 1.41, 95%CI; 0.96–2.04). Rublee et al. also found no association between chronic kidney disease and adherence (OR 1.14, 95%CI; 0.97–1.35). Finally, Aarnio et al. (n = 116846, QA score = 16) found that women (OR 0.90, 95%CI; 0.82–0.99) but not men (OR 0.94, 95%CI; 0.83–1.08) with rheumatoid arthritis were less likely to adhere.

### Health Behaviours & Lifestyle Factors

Eight studies evaluated health behaviours or lifestyle factors [30,33,35,41,44–47].

#### Alcohol misuse inversely associated with statin adherence—Strength of evidence = 4

Four studies evaluated this association. Two studies (n = 151140, n high quality = 2) reported that severe alcohol misuse, or alcoholism nearly doubled the risk of non-adherence after adjustment for other factors, in particular Bryson et al. were able to control for potential confounding from level of education and smoking status [34,36]. Two studies (n = 6616, n high quality = 1) found no effect of alcohol use or extreme drinking on statin adherence [45,46]. However the level of drinking captured in these studies may not be comparable to a diagnosis of alcoholism or self-reported severe alcohol misuse.

#### Physical activity does not associate with adherence—strength of evidence = 3

Halava et al. (QA = 16, n = 6458) found no relationship between the level of self-reported activity measured using the metabolic equivalent of task (MET) index and adherence to statins either unadjusted or adjusted analyses [46]. Accurately capturing physical activity via self-report is challenging and further research using objective methods would better determine if there is a link between adhering to statins and exercise.

#### The relationship between dietary behaviours and statin adherence—Strength of evidence = 0

Two studies Guthrie (QA score = 5, n = 4548) and Mann et al (2007) (QA score = 14, n = 71) reported contradictory findings on participants’ dietary behaviours and adherence [47,48].

#### Health seeking behaviours associated with adherence—Strength of evidence = 3

Three studies [42,47,48] contained information on other types of health seeking behaviours. In two studies seeing a general practitioner increased the odds of being adherent by 20 to 25% [42,48]. Mann et al. reported that visiting a healthcare practitioner for a cholesterol check increased the odds of being adherent by a factor of four [46]. Rublee et al. (N = 79010, QA score = 13) captured data on the number of people who attended clinic for preventive services [41]. These included vaccinations, screening tests for bowel cancer, screening for osteoporosis, cervical and breast cancer in women and prostate screening in men. The size of the association varied depending upon the preventive service used but unadjusted analyses revealed people who undertook these health seeking behaviours were 10 to 30% more likely to be adherent during the one year adherence assessment period.

#### Other health behaviours associate with statins adherence—Strength of evidence = 1–2

Guthrie et al investigated the association between other self-reported health behaviours and adherence to statins (N = 4548, QA score = 5) [48]. Self-reported use of blood pressure control treatments, trying to quit smoking and increased physical activity increase the odds of being adherent by 40 to 50%. These were all unadjusted analyses and the study was deemed low quality, therefore extrapolation of these results to the primary prevention population in general is limited.

#### Health beliefs associate with adherence—Strength of evidence = 2

Mann et al. (N = 71, QA score = 14) investigated the association between health beliefs and adherence using a bespoke health belief questionnaire. Within the study there were conflicting findings (Table 3). Participants who endorsed the beliefs “plan to use statin: < 6 months”, “personal risk of a heart attack: less than average”, and “statins may be harmful to me” were two to three times less likely to adhere than veterans who did not endorse these views [47].

### Treatment-related predictors

#### Polypharmacy is not associated with statin adherence—Strength of evidence = 3

Four studies investigated the association between the total number of medications a person received and adherence to statins [32,34,39,45]. Aarnio et al (QA score = 16, N = 116846) found that after adjustment for all other variables there was no increased likelihood of adherence per additional medicine for men and women [34]. Lavikainen et al. (QA score = 13, N = 42807) used a subsample from the same female cohort as Aarnio et al. but categorised the total number of medications into groups of 1–2, 3–5 and 6–31 medications. In unadjusted analyses patients who took more medications were 10 to 20% more likely to be adherent [39]. Farsaei et al reported adherent patients took 1.4 more medications than non-adherent patients (QA score = 7, n = 158) [45], and Braamskamp et al. found no association after adjusting for other factors between using other medications (yes v no) and adherence in their young familial hypercholesterolemia cohort (QA score = 13, N = 169) [32]. It is likely people on statins are prescribed other preventive CVD treatments such as antihypertensive therapies.

#### Type of statin associates with adherence—Strength of evidence = 3

Four studies examined the type of statin and the direction and size of effect for particular statins varied greatly across studies [31,34,37,40]. Given the high likelihood of bias from indication for particular statins only Aarnio et al. is reported here because these analyses adjusted for potential confounders (Table 3). Compared to simvastatin, people were more likely to adhere to fluvastatin and rosuvastatin, and less likely to adhere to lovastatin. After adjustment for other factors, Finnish people using pravastatin were no more adherent than people who used simvastatin [34].

#### Intensity of statin dose inversely associated with adherence = strength of evidence = 3

Aarnio et al. set cut-offs of intensity for each type of statin and then classed people as having a low, moderate or high dose of statins. Men on a moderate daily dose (OR 0.89, 95%CI;0.84–0.94) and men on a high daily dose were less likely to adhere compared to men on a low daily dose of statins (OR 0.70, 95%CI; 0.54–0.92) [34]. Similar and larger effects were observed in the cohort of women, women on a high daily dose of statins were 60% less likely to adhere compared to women on a low daily dose.

#### Timing of statin initiation associates with adherence = strength of evidence = 2

Lavikainnen et al (QA score = 13, N = 42805) collected the year that the statin was initiated and, compared to the year 2001, the proportions of women who were classed adherent were higher for the years 2002, 2003 and 2004 [39]. In a cohort of patients (QA score = 13, N = 169) with familial hypercholesterolemia, those who initiated statins before puberty were no more likely to be currently adherent than those who were first prescribed statins post puberty [32].

#### Longer pharmacy prescription associates with statin adherence = strength of evidence = 3

Batal et al. (QA score = 15, N = 3386) demonstrated that a longer supply of statins, 60 versus 30 days, was associated with an increased likelihood of being adherent after adjustment for clinical and demographic factors (RR 1.40, 95%CI;1.27–1.55) [35].

#### Other treatment related factors = strength of evidence = 1–2

Al-Foraih reported an unadjusted positive effect of longer statin duration on adherence; however, the authors did not describe over what period of time this was measured, and this study may be susceptible to left censorship [31]. Finally one large high quality study captured data on which professional had prescribed the statin [43]. People were nearly four times more likely to be adherent if the initial prescriber was a cardiologist than if the prescriber was a general practitioner, but this was an unadjusted analysis, without adjusting for the number of CVD risk factors. This effect is likely to be confounded by factors that influence whether the patient has a consultation with a cardiologist rather than a general practitioner. Braamskamp et al. also reported that self-reported side effects did not associate with adherence but noted that a minority of the cohort reported side effects.

#### Medication costs association with adherence—Strength of evidence = 0

Two studies using three different measures evaluated the impact of medication cost [34,35]. Aarnio et al. 2016 calculated the total out of pocket prescription costs for all medications and secondly by calculating the specific co-payment patients made with respect to their first statin prescription [34]. For every 50 euro increase in total costs there was approximately 10% increase in the likelihood of adhering. However, men and women who paid the highest tariff were over two times more likely to non-adhere than those paying the lowest tariff. The analyses adjusted for other socioeconomic factors such as income and education that may have confounded the association between cost and adherence. One other study investigated co-payments and found making co-payments (yes v no) did not affect adherence after adjustment for other factors [35], however given that 80% of this cohort made some kind of co-payment a comparison akin to Aarnio which compared different levels of co-payment may have demonstrated a difference. Given the heterogeneity of how cost was considered in the studies drawing a firm conclusion on the impact of cost is difficult.

#### No association between type of healthcare organisation and statin adherence—Strength of evidence = 3

Three studies investigated the type of healthcare delivery system and adherence [35,42,43]. Slejko et al and Batal et al. extracted data from US drug registries and investigated if the type of health plan associated with being adherent (Total N = 793396, N high quality = 1). Batal et al. compared those with or without insurance cover and found those with insurance cover were no more likely to be adherent than those without, after adjustment for other factors [35]. Data from Slejko et al. in unadjusted analyses indicate that members of commercial health insurance plans were no more likely to be adherent than those covered by Medicaid or Medicare insurance plans [43]. One study (N = 11126, QA score = 13) did report different rates of adherence for different healthcare organisations, based on the type of cover [42]. Compared to people with point of service (POS) plans, which allow people to access any healthcare professional at the point of service without paying into a plan, people with indemnity cover which does require regular payments but pays out when sickness occurs were twice as likely to be adherent. People with POS plans that limited who they could see in the healthcare service were twice as likely to be non-adherent. However these were unadjusted analyses that did not account for the different characteristics of people who use comprehensive indemnity insurance and those using basic insurance provided by the state, such as income and education level.

### Reasons for statin non-adherence

Farsaei et al. surveyed reasons for non-adherence in a diabetes sample [45]. The authors reported 50% of 158 patients forgot to take their medications, the other reasons given were: side effects (15%), because they did not take medications outside of the home (15%), because they had run out (10%) and because they had achieved their therapeutic goal (10%). Guthrie et al. reported reasons for discontinuation of pravastatin in their sample which included patient decision (2%), side effects (5%), cost (4%), physician decision (3%), switched to other medication (4%) other (6%) [48]. Harrison et al. (N = 98 QA score = 4) conducted telephone interviews with people 12 weeks after their first statin prescription and found that only 26% had filled their primary prescription [33]. Reasons for primary non-adherence included; general concerns about medication (63%), decided to modify lifestyle instead (63%), fear of side effects (53%), statin unnecessary (39%), low perceived illness severity (35%), fear of drug interactions (16%), concerns about overuse of medications (16%), financial hardship (12%), did not understand why provider prescribed medication (11%), did not understand purpose of medication (8%), did not think statins were effective for condition (7%), inconvenient dosing regimen (4%), and change in health plan (3%).

## Discussion

This is the first systematic review specifically focused on predictors of adherence to statins in the primary prevention setting. In total nineteen studies were included, and many more could have been included if results had been stratified by primary and secondary prevention. This was not a review of adherence rates overall, but the level of adherence in these reviewed studies was sub-optimal, and further emphasises the importance of focusing on improving adherence in the primary prevention population. There was moderate to strong evidence that individuals with traditional CV risk factors have better adherence. In particular older age, male gender, a diagnosis of diabetes, and a diagnosis of hypertension predicted better adherence. In contrast, the evidence that adherent patients adopt other healthy behaviours to protect their heart was limited; only the evidence for an inverse relationship between alcohol misuse and obesity (in women) and adherence was convincing. These findings challenge the concept of the “healthy adherer effect”, since those who are more ill appear to adhere to statins better. These findings are predicted by the need-concern framework of health beliefs which postulates those who perceive the greatest need despite medication concerns are more likely to adhere [53].

There was moderate to strong evidence that socioeconomic predictors such as wealth, employment status and level of education associate with statin adherence, and these effects have been observed in systematic reviews that have included primary and secondary prevention cohorts combined [20,21]. The association between higher socioeconomic status and adherence may be related to fewer practical barriers to adhering. However in this review factors such as longer prescriptions fills, medication costs and type of healthcare provision, in contrast to previous reviews that included secondary prevention populations, were inconsistently associated with statin adherence. The type and intensity of statin dose independently associated with statin adherence [34]. Whether these associations are underpinned by medication concern, such as those reported by Mann et al. or increased levels of adverse events is unknown. Only one study examined the relationship between side effects and non-adherence and returned a null finding [32]. However side effects were given as a reason for both non-commencement [33] and non-adherence [45] in two small studies, therefore this merits further investigation.

These findings align with results from previous reviews that increased health risks and male gender associate with better adherence [20,21]. In this review most studies reported a positive linear relationship between age and adherence. However, previous reviews indicate that age is nonlinearly related to adherence, suggesting age is an important modifier of the relationship between perceived risk of CVD and adherence.

In this review the association between higher income and better adherence was much clearer for men than women, and higher levels of education associated with lower statin adherence in women and higher statin adherence in men. The apparent sex dependent effect of socioeconomic status upon adherence was discussed by Aarnio et al. and they cite the unmeasured association between unhealthy lifestyles and low health literacy as an explanation [34]. There was strong evidence from this review that excess alcohol consumption in men and women was associated with lower adherence to statins, and strong evidence for women but not men with obesity to be less adherent. These associations may be partly underpinned by low health literacy. Educated men and women are likely to be more health literate. Men and women may perceive their susceptibility to primary CVD differently because sex informs the calculation of CVD risk. Alternatively, men and women may balance the overall need for a preventive medication with medication concerns differently.

These data suggest that individuals who are younger, female, or do not have diabetes or hypertension may have a lower perceived risk of developing CVD or experiencing a CVE [54], and reminders alone may not be sufficient to change behaviour [55]. The very limited evidence from this review supports the hypothesis that low perceived susceptibility to CVD independently predicted poor adherence [47]. Interventions that aim to improve patient and/or physician understanding of personal risk of incident CVD have demonstrated improved statin adherence in the short-term [56,57]. Lower risk individuals may also have less contact with medical professionals and there was limited evidence that attending clinic, or having a cholesterol check associated with better statin adherence.

### Methodological limitations

The findings of this review must be interpreted in the context of its limitations. What constitutes a primary prevention population was fairly consistent across studies. One study excluded patients with diabetes and hypertension as Halava et al. considered these CVD risks may modify the relationship between lifestyle factors and adherence [46]. Data pertaining to potential predictors were extracted from ten studies to calculate univariable associations between these factors and statin adherence. The interrelatedness of clinical, socioeconomic and lifestyle factors cannot be addressed in such analyses. Where studies conducted multivariable analyses with large numbers of variables collinearity may not have been sufficiently addressed. Further research is therefore required to understand the relationship between the presence of cardiovascular risks, adoption of healthy lifestyles and statin adherence.

A limitation of measuring adherence is the risk of measurement reactivity; this was recently demonstrated in two RCTs designed to improve adherence. Patients were screened for sub optimal adherence based on their pharmacy refill records before entry in to the trial. These trials objectively measured adherence using a MEMS. After 3 months the level of adherence was very high irrespective of treatment arm with no significant differences. Given that only 1% of the sampling frame agreed to participate in these trials and the median patient adherence before entry into these trials was ~60% it appears that the effective intervention to optimise adherence involved the patient’s decision to participate in these studies, and potentially the patient’s response to the electronic monitoring of their medication [58]. However, most of the studies included in this review used objective indirect methods to assess adherence, meaning the participant was unaware that they were being monitored. Therefore, there is limited bias in these data from measurement reactivity. Prescription refill data still have limitations both because there is an underlying assumption that all prescribed pills were taken between fills and because it is not possible to identify periods of time when a prescription is not filled because of medical advice to temporarily stop a statin.

Fixed dose combination (FDC) therapy or “polypill” therapies that combine blood pressure, cholesterol lowering and antiplatelet treatments into a single pill, are hypothesised as one strategy to improve adherence. The authors did not include FDC trials in this review because adherence to a polypill necessarily cannot be specific to a statin. Adherence to FDC compared to multiple CVD medications was investigated as part of a Cochrane review and levels of adherence were higher in the FDC arm but this was investigated in just one study [59]. In our review, four studies investigated polypharmacy and there was no evidence that polypharmacy lowered adherence to statins; one study reported that adherence increased per additional medication. Similar inconsistent effects have been observed in previous reviews. Given the current evidence base, FDC therapy is unlikely to increase adherence to statins. Other features of medicine use (time taken to complete, regimen complexity) may have a greater impact on long-term adherence than simply the number of medications.

## Future research

This review draws attention to the limited number of well-designed observational studies examining multivariable predictors of adherence. Synthesis of the existing data allows one to infer possible mechanisms but there are a number of areas where more research is required. Firstly, is high perceived risk of primary CVD the mechanism that explains the observed associations between traditional CVD risk factors and statin adherence? Secondly, given there is evidence of sex dependent effects of socioeconomic factors on adherence, what are the drivers of these sex differences? Potentially, there is an interaction between gender and level of health literacy which results in gendered beliefs about the need for and concerns about statins, but this still needs demonstrating empirically. Thirdly, given that side effects and fear of side effects were given as reasons for discontinuation, it is remarkable that only one study investigated the relationship between side effects and adherence [32]. Patient tolerance of side effects could explain the observed links between dosing and type of statin and adherence [34], alternatively, people with prior negative expectations may misattribute symptoms such as muscular pain (myalgia) to statins use [60]. The low level of reporting of adverse events prevents the authors from drawing any conclusion about the effect of side effects on statin adherence. Therefore, future research should address the relationship between prior concerns about side effects, reported side effects and statins adherence. Fourthly, the relationship between lifestyle factors and statins adherence is poorly understood, nevertheless there is some evidence these associations may also be modified by patient factors. Future analyses using high quality prospective data could investigate if gender and age modify the association between lifestyle factors and adherence. Such analyses would allow one to infer the possible drivers of these differences. For example, high physical activity in a middle aged woman may predict poor statin adherence, because this woman perceives herself to be at low risk of disease, whereas high physical activity in an older man may predict high adherence because both his behaviours are underpinned by a high perceived risk of personal morbidity/mortality. Similarly, data from studies that investigate if the presence or absence of diabetes and/or hypertension alters the relationship between lifestyle factors and statins adherence could be used to infer the latent effects of health beliefs.

## Conclusion

There is an ongoing debate about how widespread the use of statins should be in the field of primary prevention. Hence, improving adherence should not be at the expense of supporting people to make healthy lifestyle changes. This review makes clear the predictors of adherence common to primary and secondary prevention settings. There also appear to be important sex and age dependent differences that are specific to adherence to statins prescribed for the primary prevention of CVD. Further research is needed to understand better the underlying mechanisms of statin adherence.

## Supporting information

S1 Table Literature search strategy. (DOCX)

S2 Table The Quality assessment (QA) Tool used to assess the quality of included articles. (DOCX)

S1 File PRISMA 2009 checklist. (DOC)
