# Supplementary material for: Systematic review of the predictors of statin adherence for the primary prevention of cardiovascular disease
Source: PLoS One. 2019 Jan 17;14(1):e0201196. doi: 10.1371/journal.pone.0201196 (PMC6336256; doi:10.1371/journal.pone.0201196)
Supplement: S1 Table — (DOCX) [file pone.0201196.s001.docx]

**Table S1 Literature Search strategy**

|  | **Search terms** |
| --- | --- |
| **1** | (statin or atorvastatin or cerivastatin or fluvastatin or lovastatin or pravastatin or simvastatin or or lipitor or baycol or lescol or mevacor or altocor or pravachol or lipostat or zocor or mevinolin) |
| **2** | (compactin or fluindostatin or rosuvastatin or dalvastatin or altocor or pravachol or lipostat or zocor or mevinolin) |
| **3** | (medostatin or mevinacor or livalo or pitava or pitavastatin or pravasin or mevalotin or gerosim or lipex or zenas or crestor or meglutol) |
| **4** | 1 or 2 or 3 |
| **5** | (randomized controlled trial or controlled clinical trial or randomized controlled trials or double blind or single blind or experimental) |
| **6** | (((observational or prospective or retrospective or factors or predictors or case control or cohort or cross sectional) not case report) or case series) |
| **7** | 5 or 6 |
| **8** | (adherence or compliance or medication refusal or treatment refusal) |
| **9** | (missed medication or missed treatment or resisted medication or resisted treatment) |
| **10** | (discontinuation or persistence) |
| **11** | (nonadherence or non-adherence or noncompliance or non-compliance or non adherence or non compliance) |
| **12** | 8 or 9 or 10 or 11 |
| **13** | (Human$ not animal$) |
| **14** | 4 and 7 and 12 and 13 |
| **15** | (discontinuation medicine or discontinuation treatment or persistence medication or persistence treatment) |
| **16** | 8 or 9 or 11 or 15 |
| **17** | 4 and 7 and 13 and 16 |
| **18** | limit 17 to yr="1984-Current" |
| **19** | remove duplicates from 18 |
| **20** | limit 19 to English language |
