# Supplementary material for: Systematic review of the predictors of statin adherence for the primary prevention of cardiovascular disease
Source: PLoS One. 2019 Jan 17;14(1):e0201196. doi: 10.1371/journal.pone.0201196 (PMC6336256; doi:10.1371/journal.pone.0201196)
Supplement: S2 Table — (DOCX) [file pone.0201196.s002.docx]

Table S2 The Quality Assessment (QA) Tool used to assess the quality of included articles.

| **Quality assessed** | | **Coding framework** | |  |
| --- | --- | --- | --- | --- |
| Appropriate methods to select participants | |  |  |  |
| 1 | Sampling frame, age and sex of sample described. | yes | no | don't know |
| 2 | >80% participation or comparison of consents and refusals | yes | no | don't know |
| Appropriate methods to measure adherence | |  |  | don't know |
| 3 | Measure of adherence reproducible | yes | no | don't know |
| 4 | Adherence Measure | Objective | Validated | Unvalidated |
|  | |  | subjective | subjective |
| Appropriate Measure of outcome | |  |  |  |
| 5* | Measure of outcome/predictor reproducible | yes | no | don't know |
| 6* | Objective measure of outcome/predictor | yes | no | don't know |
| Appropriate methods to reduce bias | |  |  |  |
| 7 | Recall period less than 2 weeks | yes | no | don't know |
| 8 | Consecutive, stratified, or randomised sample | yes | no | don't know |
| Appropriate methods to reduce confounding | |  |  |  |
| 9* | Confounding controlled for in analysis | yes | no | don't know |
| 10* | Confounding quantified in analysis | yes | no | don't know |
| Appropriate methods to report adherence statistics | |  |  |  |
| 11 | Adherence reported with a measure of dist. | yes | no | don't know |
| Appropriate methods to analyse strength of association with outcome | | |  |  |
| 12* | Association reported with 95% CI | yes | no | don't know |
| 13* | Association accounts for skewedness | yes | no | don't know |
| 14 | N>30 = 1 | yes | no | don't know |
| 15* | 10 cases per predictor | yes | no | don't know |
| Conflict of interest | |  |  |  |
| 16 | Conflict of Interest declaration | yes | no | don't know |
